# From Nash Equilibrium to Social Optimum and Back: A Mean Field Perspective

**DOI:** 10.1007/s00245-026-10447-7

**Published:** 2026-05-13

**Authors:** René Carmona, Gökçe Dayanıklı, François Delarue, Mathieu Laurière

**Affiliations:** 1 ORFE, Bendheim Center for Finance, Princeton University, Princeton, NJ 08544 USA; 2 Department of Statistics, University of Illinois at Urbana-Champaign, Champaign, IL 61820 USA; 3 Laboratoire J.A. Dieudonné, Université Côte d’Azur, Parc Valrose, 06108 Nice, France; 4 Shanghai Frontiers Science Center of Artificial Intelligence and Deep Learning, NYU-ECNU Institute of Mathematical Sciences, NYU Shanghai, Shanghai, 200126 People’s Republic of China

**Keywords:** Mean field games, Mean field control, Mechanism design, Nash equilibrium, Social optimum

## Abstract

Mean field games (MFGs) and mean field control (MFC) have been introduced to study large populations of strategic players. They correspond respectively to non-cooperative or cooperative scenarios, where the aim is to find the Nash equilibrium and social optimum. They approximate finite player problems and have many applications in economics, biology, machine learning. This paper studies how players can pass from a non-cooperative to a cooperative regime, and vice-versa. The first direction is reminiscent of mechanism design, in which the game’s definition is modified so that non-cooperative players reach an outcome similar to a cooperative scenario. The second direction studies how initially cooperative players gradually deviate from social optimum to reach Nash equilibrium when they optimize their individual cost very much in the spirit of the free-rider phenomenon. To formalize these connections, we introduce and theoretically analyze two new classes of games which lie between MFG and MFC: $\lambda$-interpolated MFGs, in which the cost of an individual player is an interpolation of the MFG and the MFC costs, and *p*-partial MFGs, in which a proportion of the population deviates from social optimum by behaving non-cooperatively. We conclude by providing an algorithm for myopic players to learn a *p*-partial mean field equilibrium.

## Introduction

### Background and Motivations

Game theory provides a rigorous framework for studying interactions among multiple agents, also called players, who make strategic decisions to optimize certain objectives. Games can be either *static* (i.e., without a time component) or *dynamic* (involving discrete or continuous time). In this work, we focus on *continuous-time stochastic games*. For background on this class of games, we refer to [1–4] and [5, Chapter 16]; for applications in operations research, see, e.g., [6–11]. The players may interact in non-cooperative, cooperative, or mixed settings, depending on the application area. Non-cooperative interactions are typically analyzed using the notion of *Nash equilibrium*, where each agent minimizes their own cost. Cooperative interactions, on the other hand, are usually studied through the notion of a *social optimum*, where players jointly minimize an average cost over the population.

As the number of players increases, exact solutions to such games become intractable. *Mean field approximations* provide a framework to find approximate equilibria or social optima in large-population games with homogeneous players that interact symmetrically, and the quality of the approximation improves as the number of players grows. In this direction, *mean field games (MFGs)* have been introduced to approximate non-cooperative situations, while *mean field control (MFC) problems* approximate cooperative settings or, alternatively, situations in which a social planner prescribes socially optimal behaviors to players. For the original works on MFGs and MFC, we refer to [12–14]. Various examples of finite-population games that can be approximated by MFGs are presented in [15, Chapter 1], while rigorous approximation results can be found in [16, Chapter 5] and [17, Chapter 6]. MFGs and MFC have in particular attracted the attention of the operations research community, with several recent developments motivated by real-world applications, such as singular or impulse controls [18, 19], games with ranking or relative performance criteria [11, 20, 21], robustness analysis [22], Stackelberg equilibria [23, 24], portfolio liquidation [25, 26], network games [27–29], learning algorithms [30, 31], or games with correlated equilibria [32, 33].

In many applications of game theory, and particularly in *mechanism design* or *policy making* for large populations, it is typically assumed that players interact in a non-cooperative manner, each optimizing their own objective while accounting for the aggregate effect of others. In this setting, the relevant solution concept is the *Nash equilibrium*, where no player can unilaterally improve their outcome by changing their strategy. By construction, Nash equilibria are stable: players have no incentive to deviate. However, they are often *inefficient* from a global perspective, as the resulting outcome may be suboptimal compared to a solution that minimizes a *social cost*, either through altruistic behavior or centralized coordination by a social planner.

This inefficiency is a central concern in both theoretical and applied settings. A well-known example is the Braess paradox (see, e.g., [34]), which illustrates how individual optimization can degrade overall system performance. To quantify this phenomenon, several notions have been introduced, including the *Price of Anarchy (PoA)* and the *Price of Stability (PoS)*, which compare the cost of Nash equilibria to that of the social optimum. PoA was first proposed in [35] and later computed for specific routing games in [36]. Extensions to dynamic settings include deterministic differential games [37], linear-quadratic MFGs [38], and models of congestion in crowd motion [39, Sect. 4.4].

In many systems involving large populations such as electricity markets, epidemic mitigation, or environmental regulation, players act non-cooperatively and independently, often leading to outcomes that are socially suboptimal. A central challenge in such settings is how to incentivize individuals to behave in a way that aligns with the social optimum, without requiring cooperation or centralized control. This motivates a line of inquiry inspired by mechanism design: *Can one design cost modifications – interpreted as taxes, subsidies, or penalties – that induce a non-cooperative Nash equilibrium whose outcome coincides with that of a socially optimal solution?* For example, one can modify the cost functions of electricity producers in a free market by introducing subsidies, thereby guiding them toward socially optimal outcomes while preserving the non-cooperative structure of the market. Crucially, this approach differs from Stackelberg MFGs or classical contract theory, where a regulator optimizes their own utility; here, the goal is to shape the incentives so that self-interested players still reach the socially desired behavior. This is particularly relevant in regulating *Tragedy of the Commons* scenarios [40], where individual incentives lead to over-exploitation of shared resources, but appropriate cost design may sustain cooperation-like outcomes. Additionally, in many real-world systems, abrupt changes in policy are impractical or undesirable, so one may seek gradual or smooth interpolations between individualistic and cooperative objectives. An equally important and related question is understanding the instability of social optima under unilateral deviations. This instability can give rise to the *free-rider* phenomenon, where some players benefit from the cooperative efforts of others without contributing themselves. Unlike Nash equilibria, social optima are not self-enforcing: individual players have an incentive to deviate if they expect others to behave cooperatively. Quantifying this instability helps explain why non-cooperative behavior often persists, even when social cooperation would be globally beneficial.

These considerations motivate the study of how mean field populations can be guided to transition between non-cooperative (Nash equilibrium) and cooperative (socially optimal) regimes, and how the inefficiencies of Nash equilibria in large populations can be understood, quantified, and potentially mitigated through incentive design.

### Literature Review and Related Work

#### Mean Field Games, Mean Field Control, and Their Connections

MFGs were introduced independently and simultaneously by Lasry and Lions [41, 42] and by Huang et al. [13] as a tractable framework for analyzing Nash equilibria in games with a large number of players. In the MFG framework, the number of players is taken to be infinite, with players assumed to be indistinguishable, negligible, and interacting symmetrically. This allows one to study a representative agent whose behavior is coupled to the population through the distribution of states. To characterize the MFG Nash equilibrium, the aforementioned original works used a system of coupled forward–backward partial differential equations, namely, the Hamilton–Jacobi–Bellman (HJB) and Kolmogorov–Fokker–Planck (KFP) equations. Subsequent developments introduced probabilistic formulations for both MFG equilibria and socially optimal solutions in MFC problems; comprehensive treatments are available in [15, 17]. As mentioned above, the inefficiency of MFG equilibria compared to socially optimal behavior is typically measured via the PoA, defined in the mean field setting as the ratio of the worst-case expected cost in a MFG equilibrium to the expected cost under the MFC solution. By definition, the PoA is always greater than or equal to 1. In certain applications, the equilibrium can be efficient: for instance, [43] show that the MFG equilibrium in an electric vehicle charging problem achieves PoA equal to 1. In a different direction, [44] demonstrate that under specific structural conditions, the solution to a standard MFG coincides with the solution to a modified social planner’s problem. This differs from our approach: in the first part of our paper, we construct an *incentivized* MFG whose equilibrium coincides with the solution to the *original* MFC problem, thereby directly aligning non-cooperative behavior with the social optimum.

#### Mechanism Design and Incentivization in Large Games

In the economics literature, the question of incentives and mechanism design has attracted a significant interest, see, e.g., [45, 46]. In the MFG literature, incentivization is often modeled by introducing a principal or regulator who interacts with the population and has a distinct objective from that of the players. This setup is commonly studied through the lens of contract theory, where the principal designs contracts to influence the behavior of non-cooperative players. For example, [23, 47, 48] analyze principal-agent problems in which a principal contracts with a mean field population of strategic players. In a related setting, [49] considers a contract theory framework involving a government and a fully cooperative population. Such problems are also referred to as Stackelberg MFGs. An application to carbon emissions is studied in [50] and a computational approach to solving Stackelberg MFGs using a single-level formulation is proposed in [24]. The design of incentives in the context of Stackelberg MFG has been studied in [51]. As noted earlier, the mechanism design approach we take in this paper differs fundamentally from the Stackelberg or contract-theoretic frameworks. We do not introduce a principal with their own objective who solves a separate optimization problem in a leader-follower setting. Instead, we focus on designing incentive modifications to the cost functions of the players themselves (within a decentralized, non-cooperative framework) so that the resulting MFG equilibrium aligns with the social optimum of the original MFC problem.

#### Mixed Populations in Mean Field Models

In [52], where the authors propose an extension of MFGs in which each player solves an MFC problem. Such games can be interpreted as the mean field limit of competition among a large number of large coalitions. In [53], the authors introduce linear-quadratic MFGs, where players factor in others’ costs and rewards (positively or negatively) when making decisions, they call these models *co-opetitive* linear-quadratic MFGs. More recently, [54] formulated a bi-level optimization problem aimed at balancing equilibrium and social optimum. The MFG literature also includes models with multiple populations, where players within each population are either non-cooperative [55, 56] or cooperative [57, 58]. These are commonly referred to as multi-population MFGs and mean field type games, respectively; see, for example, [59] for a comparison. In contrast to our setup, however, players in these models are assigned fixed types (cooperative or non-cooperative) and do not switch behavior. Recently, the cooperation and competition mixture in MFGs at the individual and population level with a focus on addressing the tragedy of the commons and common pool resource modeling has been studied in [60, 61].

### Contributions and Paper Structure

This paper bridges the gap between Nash equilibria in MFGs and socially optimal solutions in MFC by addressing when and how a population of non-cooperative players can be incentivized to behave as if they were cooperating and how the unstability of socially optimal solutions can result in changes in the behavior. Our first contribution is the introduction of a flexible framework to embed incentives directly into players’ cost functions, including the proposal of a family of $\lambda$-*interpolated MFGs*, which smoothly transition between purely selfish and purely cooperative behavior. We establish *well-posedness* and *continuity* results for these models. Second, we quantify the fragility of social optima by introducing the *Price of Instability*, a new metric that measures how strongly a single agent is tempted to deviate from a cooperative plan. Third, we introduce and analyze the *p*-*partial mean field equilibrium*, which models settings where a known fraction of the population deviates unilaterally. We establish *well-posedness* and *continuity* results for this model. We show how the associated free-rider effects degrade the social cost and characterize the equilibrium structure. Finally, we investigate a dynamic adjustment process in which players, lacking full knowledge of the population, gradually deviate from cooperation through myopic updates, and we prove convergence to a *p*-partial equilibrium in this learning regime.

The paper is organized as follows. Section 2 introduces the mean field setting and formulates the MFG and MFC problems. Section 3 studies how to design incentives that align non-cooperative behavior with socially optimal outcomes, including two approaches: matching the social cost value (Sect. 3.1) and replicating the social planner’s optimal control (Sect. 3.2). Section 4 investigates the instability of social optima under unilateral deviations. We first define the Price of Instability for single-player deviations (Sect. 4.1), then introduce the *p*-partial MFG for known proportions of deviators (Sect. 4.2), and finally analyze a myopic adjustment process in a learning setting (Sect. 4.3). Some proofs and discussions are presented in Appendices 1 and 1. We provide a list of notations in Appendix 1.

## The Model

In this section, we first introduce notations used throughout the paper and present the model we consider. We then introduce the MFG and the MFC problems.

### Notation

The space of probability measures on $\mathbb {R}^d$ with a second moment is denoted by ${\mathcal {P}}_2(\mathbb {R}^d)$ and endowed with the 2-Wasserstein distance (see e.g., [15, Chapter 5]) defined as:

$$ W_2(\mu ,\mu ') = \inf _{\pi \in \Pi (\mu ,\mu ')} \left( \int _{{\mathbb {R}}^d \times {\mathbb {R}}^d} |x-x'|^2 \pi (dx,dx')\right) ^{1/2}, $$

where $\Pi (\mu ,\mu ')$ is the set of all probability measures on ${\mathbb {R}}^d\times {\mathbb {R}}^d$ with a second moment and where the marginals are $\mu$ and $\mu '$, respectively. The collection of square-integrable and $(\mathcal {F}_t)_{t\in [0,T]}$-progressively measurable action processes $\boldsymbol{\alpha }= (\alpha _t)_{t\in [0,T]}$ where $\alpha _t \in {\mathbb {R}}^d$ is denoted by $\mathbb {A}$. In the sequel, if *X* is a random variable, we denote by $\mathcal {L}(X)$ the law of *X*.

We will consider two notions of derivatives with respect to measures. The first notion is the *Lions derivative*, which we denote by $\partial _\mu$. We recall here the definition and refer to e.g., [62] or [15, Chapter 5] for more details. A function $U: {\mathcal {P}}_2({\mathbb {R}}^d) \rightarrow {\mathbb {R}}$ is differentiable if there exists a map $\partial _\mu U: {\mathcal {P}}_2({\mathbb {R}}^d) \times {\mathbb {R}}^d \rightarrow {\mathbb {R}}$ such that for any $\mu ,\mu ' \in {\mathcal {P}}_2({\mathbb {R}}^d)$,

$$ \lim _{s \rightarrow 0^+} \frac{U((1-s)\mu + s\mu ') - U(\mu )}{s} = \int _{{\mathbb {R}}^d} \partial _\mu U(\mu , x') d(\mu ' - \mu )(x'). $$

We say that *U* is $\mathcal {C}^1$ if $\partial _\mu U$ is continuous. If *X* is an ${\mathbb {R}}^d$ random variable and $\varphi : {\mathbb {R}}^d \times {\mathbb {R}}^d \rightarrow {\mathbb {R}}$, then the notation $\tilde{{\mathbb {E}}}[\varphi (X, {\tilde{X}})]$ means that the expectation is taken (only) over $\tilde{X}$, which is an independent copy of *X*.

The second notion is the functional derivative. We borrow the definition from [15, Definition 5.43]. A function $U: {\mathcal {P}}_2({\mathbb {R}}^d) \rightarrow {\mathbb {R}}$ is said to have a *functional* (or *flat* or *linear*) *derivative* if there exists a function

$$ \frac{\delta U}{\delta m}: {\mathcal {P}}_2({\mathbb {R}}^d) \times {\mathbb {R}}^d \ni (\mu , x) \mapsto \frac{\delta U}{\delta m}(\mu )(x) \in {\mathbb {R}}, $$

continuous for the product topology, such that for any subset ${\mathcal {K}}\subseteq {\mathcal {P}}_2({\mathbb {R}}^d)$, the function ${\mathbb {R}}^d \ni x \mapsto [\delta U/ \delta m](\mu )(x)$ is at most of quadratic growth in *x* uniformly in $\mu \in {\mathcal {K}}$, and such that for all $\mu$ and $\mu '$ in ${\mathcal {P}}_2({\mathbb {R}}^d)$, it holds:

$$ U(\mu ') - U(\mu ) = \int _0^1 \int _{{\mathbb {R}}^d} \frac{\delta U}{\delta m}(t\mu ' + (1-t)\mu )(x) d[\mu ' - \mu ](x) dt. $$

Notice that $\frac{\delta U}{\delta m}$ is not defined uniquely but it is unique up to an additive constant. For the sake of definiteness, unless otherwise specified, we will assume that the following normalization condition is satisfied: $\int \frac{\delta U}{\delta m}(\mu )(x) \mu (dx) = 0$. This selects a unique function for $\frac{\delta U}{\delta m}$. However, the choice of this constant (0 or another value) is not going to impact our analysis. The two notions of derivatives are related by the formula:

1 $$\begin{aligned} \partial _x\frac{\delta U}{\delta m}(\mu )(x)=\partial _\mu U(\mu )(x), \qquad (\mu , x) \in {\mathcal {P}}_2({\mathbb {R}}^d) \times {\mathbb {R}}^d. \end{aligned}$$

We refer to [15, Chapter 5] and [62] for more details on the connection between the two notions of derivatives. In particular, the two notions are not equal in general, see e.g. [15, Chapter 5, Example 1] for a counterexample.

### Model and Assumptions

#### Model

Let $T > 0$ be a finite time horizon. We assume that the problem is set on a complete filtered probability space $(\Omega , \mathcal {F}, \mathbb {F} = (\mathcal {F}_t)_{t\in [0,T]}, \mathbb {P})$ supporting a *d*-dimensional Wiener process $\boldsymbol{W}= (W_t)_{t\in [0,T]}$ that represents the idiosyncratic noise. The representative player has a continuous ${\mathbb {R}}^d$-valued state process $\boldsymbol{X}= (X_t)_{t\in [0,T]}$. In the rest of the paper, we consider the following mean field model. Let $\mu _0$ be an initial distribution. The state process $\boldsymbol{X}^{\boldsymbol{\alpha }}$ for a representative player has the following dynamics:

2 $$\begin{aligned} \begin{aligned} dX_t^{\boldsymbol{\alpha }}=\alpha _t dt + \sigma dW_t, \quad t \in [0,T], \quad X_0^{\boldsymbol{\alpha }} \sim \mu _0, \end{aligned} \end{aligned}$$

where $\sigma \in {\mathbb {R}}^{d\times d}$ is a non-zero constant matrix. For each fixed flow of probability measures $\boldsymbol{\mu }=(\mu _t)_{0\le t\le T}$, the representative player has the cost:

3 $$\begin{aligned} J^{\boldsymbol{\mu }}(\boldsymbol{\alpha }) = J(\boldsymbol{\alpha }; \boldsymbol{\mu })=\mathbb {E}\left[ \int _0^Tf\left( t,X_t^{\boldsymbol{\alpha }},\mu _t,\alpha _t\right) dt + g\left( X_T^{\boldsymbol{\alpha }},\mu _T\right) \right] , \end{aligned}$$

where $f: [0,T]\times {\mathbb {R}}^d \times {\mathcal {P}}_2({\mathbb {R}}^d) \times {\mathbb {R}}^d \rightarrow {\mathbb {R}}$ is the running cost of the representative player that depends on her state $X_t^{\boldsymbol{\alpha }}$, the population distribution $\mu _t$ of states, and her control $\alpha _t$, and $g: {\mathbb {R}}^d \times {\mathcal {P}}_2({\mathbb {R}}^d) \rightarrow {\mathbb {R}}$ is the terminal cost of the representative player that depends on her terminal state $X_T^{\boldsymbol{\alpha }}$ and the population distribution $\mu _T$ of the states at the terminal time. When the control $\boldsymbol{\alpha }$ is clear from the context, we will omit the superscript on $\boldsymbol{X}$.

Although many of the ideas developed in the rest of the paper could be extended to more complex models, for the sake of simplicity, we consider that the running cost function *f* is of the form:

4 $$\begin{aligned} f(t,x,\mu ,\alpha )=\frac{1}{2}|\alpha |^2 + f_0(x,\mu ), \end{aligned}$$

where $|\cdot |$ denotes the Euclidean norm. We emphasize that this simplified model form has the advantage of providing clear interpretations and theoretical analysis of the two main objectives of this work: how to design incentives to drive the players behavior towards a cooperative equilibrium and explaining the free-riding phenomenon which is the case of deviations from the MFC equilibrium.

The assumptions on $f_0$ are stated below in Assumption 1. In particular, we will assume $f_0$ to be differentiable with respect to *x* and differentiable with respect to $\mu$ for both notions of derivatives that we recalled above, namely, in the Lions sense and also in the functional sense.

The (reduced) Hamiltonian $H: [0,T] \times {\mathbb {R}}^d \times {\mathcal {P}}_2({\mathbb {R}}^d) \times {\mathbb {R}}^d \times {\mathbb {R}}^d \rightarrow {\mathbb {R}}$ is defined by:

5 $$\begin{aligned} H(t,x,\mu , y, \alpha ) = \alpha \cdot y +\frac{1}{2}|\alpha |^2 + f_0 (x,\mu ). \end{aligned}$$

We will denote its minimizer by ${\hat{\alpha }}: [0,T] \times {\mathbb {R}}^d \times {\mathcal {P}}_2({\mathbb {R}}^d) \times {\mathbb {R}}^d \rightarrow {\mathbb {R}}^d$, which is:

6 $$\begin{aligned} {\hat{\alpha }}(t,x,\mu ,y) \in \mathop {\mathrm {arg\,min}}\limits _{\alpha } H(t, x, \mu , y, \alpha ) = -y. \end{aligned}$$

#### Model Assumptions

We continue with introducing several sets of assumptions on $f_0$ and *g*.

##### Assumption 1

(i) The functions $f_0$ and *g* are continuously differentiable with respect to *x* and differentiable with respect to $\mu$ (in the sense of $\partial _\mu$). Furthermore, we assume that, for any $(x,\mu ) \in {{\mathbb {R}}}^d \times {{\mathcal {P}}}_2({{\mathbb {R}}}^d)$, there exists a version of $x' \mapsto \partial _\mu f_0(x,\mu )(x')$ (resp. $x' \mapsto \partial _\mu g(x,\mu )(x')$) such that the mapping $(x,\mu ,x') \mapsto \partial _\mu f_0(x,\mu )(x')$ (resp. $(x,\mu ,x') \mapsto \partial _\mu g(x,\mu )(x')$) is continuous.
(ii) The derivatives $\partial _x f_0$ are $\partial _x g$ are Lipschitz continuous (the space ${{\mathcal {P}}}_2({{\mathbb {R}}}^d)$ being equipped with $W_2$) and the derivatives $\partial _\mu f_0$ and $\partial _\mu g$ are Lipschitz continuous with Lipschitz constant *L*. In other words, for all $x, x^\prime \in {\mathbb {R}}^d,$ $\mu , \mu ^\prime \in {\mathcal {P}}_2 ({\mathbb {R}}^d)$, and any ${\mathbb {R}}^d$-valued random variables *X* and $X^\prime$ having $\mu$ and $\mu ^\prime$ as distributions respectively, we have:
  $$\begin{aligned} \begin{aligned} {\mathbb {E}}\Bigl [ \Big | \partial _\mu f_0 (x^\prime , \mu ^\prime )(X^\prime ) - \partial _\mu f_0 (x, \mu )(X)\Big |^2 \Bigr ]&\le L\Big [|x^\prime - x|^2 + {\mathbb {E}}\Big [|X^\prime - X|^2\Big ]\Big ]\\ {\mathbb {E}}\Bigl [ \Big | \partial _\mu g (x^\prime , \mu ^\prime )(X^\prime ) - \partial _\mu g (x, \mu )(X)\Big |^2 \Bigr ]&\le L\Big [|x^\prime - x|^2 + {\mathbb {E}}\Big [|X^\prime - X|^2\Big ]\Big ] \end{aligned} \end{aligned}$$

Assumption 1 subsumes assumption **Necessary SMP** (A1)–(A2) (p. 166) and assumption **Pontryagin Optimality** (A1)–(A2) (pp. 542–543) in [15], when adapted to the model (2)–(4). The first one provides a convenient form of the Pontryagin principle for MFGs and the second one for MFC.

Throughout the article, Assumption 1 is in force. We will sometimes complement it with one or other (or both) of the following assumptions:

##### Assumption 2

At least one of the following two properties holds true:

(i) The matrix $\sigma$ is non-degenerate and, for any $R>0$, the derivatives $\partial _x f_0$ are $\partial _x g$ are bounded in the *x*-argument, uniformly with respect to the entries $\mu$ satisfying $M_2(\mu ) \le R$.
(ii) The functions $f_0$ and *g* are convex in the argument *x*, and for some $C>0$, the functions $x \in {{\mathbb {R}}}^d \mapsto x \cdot \partial _x f_0(0,\delta _x)$ and $x \in {{\mathbb {R}}}^d \mapsto x \cdot \partial _x g(0,\delta _x)$ satisfy $x \cdot \partial _x f_0(0,\delta _x) > -C(1+\vert x \vert )$ and $x \cdot \partial _x g(0,\delta _x) > -C(1+\vert x \vert )$.

Assumption 2 is used to guarantee that the stochastic Pontryagin principle, which we use repeatedly in the sequel, provides a sufficient condition of optimality, and to derive the existence of an equilibrium to the MFG under study.

##### Assumption 3

The functions $f_0$ and *g* are convex in $(x,\mu )$, convexity with respect to the measure argument being understood in the displacement convex sense, namely

$$\begin{aligned} f_0 \Bigl ( \lambda x + (1-\lambda ) x' , {{\mathcal {L}}} \bigl ( \lambda X + (1-\lambda ) X'\bigr ) \Bigr ) \le \lambda f_0 \bigl (x , {{\mathcal {L}}} (X) \bigr ) + (1-\lambda ) f_0 \bigl ( x' , {{\mathcal {L}}}( X') \bigr ) \bigr ), \end{aligned}$$

for any $\lambda \in [0,1]$, $x,x' \in {{\mathbb {R}}}^d$ and ${{\mathbb {R}}}^d$-valued square integrable random variables *X* and $X'$, with respective laws ${{\mathcal {L}}}(X)$ and ${{\mathcal {L}}}(X')$, and similarly for *g*.

In combination with Assumptions 1, 3 makes it possible to apply Theorem 6.16 (p. 550) and Theorem 6.19 (p. 559) in [15], which guarantees existence and uniqueness of a minimizer to the MFC considered in this paper.

### The Mean Field Game Problem

We start with the standard MFG definition.

#### Definition 1

An MFG solution, also called an MFG (Nash) equilibrium, is a pair $(\boldsymbol{\alpha }^\mathrm{{MFG}},\boldsymbol{\mu }^\mathrm{{MFG}})$ such that $\boldsymbol{\alpha }^\mathrm{{MFG}}$ minimizes $J^{\boldsymbol{\mu }^\mathrm{{MFG}}}(\cdot )$ defined in (3), and $\mu ^\mathrm{{MFG}}_t = {\mathcal {L}}(X^\mathrm{{MFG}}_t)$ for all $t \in [0,T]$, where $\boldsymbol{X}^\mathrm{{MFG}}$ is the solution to (2) controlled by $\boldsymbol{\alpha }^\mathrm{{MFG}}$.

For more details on the connection between the MFG solution and Nash equilibria in finite-player games, we refer the interested reader e.g. to [16, Chapter 5] and [17, Chapter 6]. Solving for a Nash equilibrium involves the search for *fixed point* of the best response function. Below, we use the stochastic Pontryagin principle to identify the MFG Nash equilibrium. Even though the Pontryagin principle only provides a necessary condition for optimality, its sufficiency holds under Assumptions 1 and 2. For a given flow $\boldsymbol{\mu }=(\mu _t)_{t \in [0,T]}$ in ${{\mathcal {P}}}_2({{\mathbb {R}}}^d)$, the control problem (2)–(4) has at least one minimizer. This has been a known result, see [63, Theorems 2.2 & 2.3], and its bibliography. The stochastic Pontryagin principle provides a necessary condition for the minimizer. See for instance [15, Theorem 3.27]. In fact, under Assumptions 1 and 2, the Pontryagin system (whose form is obtained by replacing $\mathcal {L}(X_t)$ by $\mu _t$ in the system (7) below) is uniquely solvable and thus provides a characterization of the minimizer. This result follows from the results in [64] under Assumption 2.(i) or follows from [15, Theorem 3.17] under Assumption 2.(ii). As a consequence, we deduce that, for the model given in Sect. 2 with (4), the MFG equilibrium is characterized by the solution, denoted by $(\boldsymbol{X}^\mathrm{{MFG}}, \boldsymbol{Y}^\mathrm{{MFG}}, \boldsymbol{Z}^\mathrm{{MFG}})$, of the following FBSDE of the McKean–Vlasov (MKV) type (see e.g., [15, Chapter 3.3.2]):

7 $$\begin{aligned} {\left\{ \begin{array}{ll} dX_t& = -Y_tdt +\sigma dW_t\\ dY_t& =-\partial _x f_0(X_t,\mathcal {L}(X_t)) dt + Z_t dW_t,\\ Y_T& =\partial _x g(X_T,\mathcal {L}(X_T)), \end{array}\right. } \end{aligned}$$

where the drift of $\boldsymbol{X}$ is the optimal control, see (6) evaluated at $y=Y_t$, which can be interpreted as the derivative of the representative player’s value function evaluated along the player’s trajectory when the whole population is at a Nash equilibrium. When the population is at equilibrium, her equilibrium cost is obtained by using the equilibrium control as well and yields the following cost:

8 $$\begin{aligned} J^{\mu ^\mathrm{{MFG}}}(\boldsymbol{\alpha }^\mathrm{{MFG}}) = \mathbb {E}\left[ \int _0^Tf\left( t,X^\mathrm{{MFG}}_t,\mu ^\mathrm{{MFG}}_t,\alpha ^\mathrm{{MFG}}_t\right) dt + g\left( X^\mathrm{{MFG}}_T,\mu ^\mathrm{{MFG}}_T\right) \right] \end{aligned}$$

with $\mu ^\mathrm{{MFG}}_t=\mathcal {L}(X^\mathrm{{MFG}}_t)$ with $X^\mathrm{{MFG}}_t = X^{\boldsymbol{\alpha }^\mathrm{{MFG}}}_t$ for each $t\ge 0$.

Under Assumption 2.(*i*), existence of an MFG equilibrium follows from [15, Theorem 4.32]. Under Assumption 2.(*ii*), it follows from [15, Theorem 4.53].

### The Mean Field Control Problem

We now introduce the MFC problem, also referred to as the social planner’s problem, or the MKV control problem.

#### Definition 2

An MFC solution also called MFC optimum is a minimizer of the following cost, defined for each control process $\boldsymbol{\alpha }=(\alpha _t)_{0\le t\le T}$ as:

9 $$\begin{aligned} J^\mathrm{{MFC}}(\boldsymbol{\alpha })=\mathbb {E}\left[ \int _0^Tf\left( t,X_t^{\boldsymbol{\alpha }},\mathcal {L}\left( X_t^{\boldsymbol{\alpha }}\right) ,\alpha _t\right) dt + g\left( X_T^{\boldsymbol{\alpha }},\mathcal {L}\left( X_T^{\boldsymbol{\alpha }}\right) \right) \right] \end{aligned}$$

where ${\boldsymbol{X}}^{\boldsymbol{\alpha }}$ solves (2) using the control $\boldsymbol{\alpha }$ and the running cost *f* is assumed to be in the form of (4).

An MFC problem is thus an *optimization* problem. In contrast with non-cooperative set-up of MFG, in the MFC problem players can be thought as playing cooperatively or being managed by a social planner to minimize the expected social cost. In the MFG setting, when the representative player changes behavior, the population characteristics, as captured by the flow $\boldsymbol{\mu }=(\mu _t)_t$ of probability measures, remain the same. However, in the MFC setting, we assume all the players behave similarly, and when the representative player changes their control, everyone uses the new control; therefore, the population characteristics also change. We define the social cost (per individual player) as:

10 $$\begin{aligned} J^{*}:=\inf _{\boldsymbol{\alpha }}J^\mathrm{{MFC}}(\boldsymbol{\alpha }) \end{aligned}$$

Under Assumption 1, the MFC problem has an optimal control, which we denote by $\boldsymbol{\alpha }^\mathrm{{MFC}}$, i.e.,

11 $$\begin{aligned} \boldsymbol{\alpha }^\mathrm{{MFC}} \in \mathop {\mathrm {arg\,min}}\limits _{\boldsymbol{\alpha }}J^\mathrm{{MFC}}(\boldsymbol{\alpha }) \end{aligned}$$

This follows from [63, Theorems 2.2, 2.3]. The reader can easily check that Assumptions *A* and *C* in the latter reference are satisfied under Assumption 1. In particular, the convexity condition stated in Assumption *C* follows from the fact the drift in (2) is linear in $\alpha$ and the cost in (4) is convex in $\alpha$. Theorem 6.14 in [15] can be used to infer the form of the Pontryagin principle in this situation. Uniqueness of the minimizer is known to hold true under the additional convexity condition stated in Assumption 3, in which case the symbol ‘$\in$’ in (11) can be replaced by ‘$=$’, i.e.,

$$\begin{aligned} \boldsymbol{\alpha }^\mathrm{{MFC}} := \mathop {\mathrm {arg\,min}}\limits _{\boldsymbol{\alpha }}J^\mathrm{{MFC}}(\boldsymbol{\alpha }). \end{aligned}$$

Accordingly, we denote by $\boldsymbol{\mu }^\mathrm{{MFC}}$ the corresponding flow of marginal distributions of the optimally controlled state process:

12 $$\begin{aligned} \boldsymbol{\mu }^\mathrm{{MFC}}&=\left( \mu ^\mathrm{{MFC}}_t\right) _{0\le t\le T}\quad \nonumber \\ \text {with}\quad \mu ^\mathrm{{MFC}}_t&={\mathcal {L}}\left( X^\mathrm{{MFC}}_t\right) \quad \text {and}\quad dX^\mathrm{{MFC}}_t=\alpha ^\mathrm{{MFC}}_t dt + \sigma dW_t. \end{aligned}$$

The optimal cost defined in (10) can then be expressed using the notation (3) as:

$$ J^{*}:= J^{\boldsymbol{\mu }^\mathrm{{MFC}}}\left( \boldsymbol{\alpha }^\mathrm{{MFC}}\right) . $$

By using stochastic Pontryagin maximum principle and remembering that (reduced) Hamiltonian is given in (5), we can regard the process $(Y_t^\mathrm{{MFC}}:= -\alpha ^\mathrm{{MFC}}_t)_{t \in [0,T]}$ as an adjoint process solving the Backward Stochastic Differential Equation (BSDE):

13 $$\begin{aligned} dY_t&=- \Bigl (\partial _x f_0(X_t,{\mathcal {L}}(X_t)) dt + \tilde{\mathbb {E}}[\partial _\mu f_0({\tilde{X}}_t,{\mathcal {L}}(X_t))(X_t)] \Bigr ) dt+ Z_t dW_t,\end{aligned}$$

14 $$\begin{aligned} Y_T&=\partial _x g(X_T,{\mathcal {L}}(X_T)) + \tilde{\mathbb {E}}[\partial _\mu g({\tilde{X}}_T,{\mathcal {L}}(X_T))(X_T)], \end{aligned}$$

where we recall that we use the notation $\partial _\mu$ for the Lions derivative and  for independent copies (see Sect. 2.1). In summary, using an optimal control for the MFC problem, the processes $\boldsymbol{X}$ and $\boldsymbol{Y}$ solve the following FBSDE system:

15 $$\begin{aligned} {\left\{ \begin{array}{ll} dX_t& = -Y_tdt +\sigma dW_t\\ dY_t& =-\Bigl (\partial _x f_0(X_t,{\mathcal {L}}(X_t)) dt + \tilde{\mathbb {E}}[\partial _\mu f_0({\tilde{X}}_t,{\mathcal {L}}(X_t))(X_t)] \Bigr ) dt+ Z_t dW_t,\\ Y_T& =\partial _x g(X_T,{\mathcal {L}}(X_T)) + \tilde{\mathbb {E}}[\partial _\mu g({\tilde{X}}_T,{\mathcal {L}}(X_T))(X_T)], \end{array}\right. } \end{aligned}$$

which is different from the FBSDE in (7) that characterizes the MFG equilibrium. To stress that the two solutions are different, we will denote the solution to the above system by $(\boldsymbol{X}^\mathrm{{MFC}}, \boldsymbol{Y}^\mathrm{{MFC}}, \boldsymbol{Z}^\mathrm{{MFC}})$.

#### Remark 1

Similar to the MFG case, Pontryagin principle states the necessary condition for the optimality. For having sufficiency, further conditions should be satisfied, which is guaranteed by Assumption 3. Under this assumption, the system (15) is uniquely solvable, see [15, Theorems 6.16 and 6.19] and hence characterizes the (unique) MFC equilibrium. For instance, so is the case if $f_0$ and *g* are in the form: $f_0(x,\mu ) = \int _{{{\mathbb {R}}}^d} \varphi _0(x-y) d\mu (y),$ $g(x,\mu ) = \int _{{{\mathbb {R}}}^d} \psi (x-y) d\mu (y),$ where $\varphi _0$ and $\psi$ are convex functions on ${{\mathbb {R}}}^d$ which are bounded below and with Lipschitz derivatives in *x*.

We emphasize that:

16 $$\begin{aligned} J^{*}\le J^{\boldsymbol{\mu }^\mathrm{{MFG}}}\left( \boldsymbol{\alpha }^\mathrm{{MFG}}\right) \end{aligned}$$

for all MFG equilibria $\boldsymbol{\alpha }^\mathrm{{MFG}}=(\alpha ^\mathrm{{MFG}}_t)_{0\le t\le T}$. In the literature, this relationship between the MFC optimal cost and the MFG equilibrium costs has been studied extensively, and terminology like Price of Stability (PoS) and Price of Anarchy (PoA) have been used to quantify this relationship. See for example [38, 65, 66] for measures of inefficiency in the mean field models. For concrete examples and numerical illustrations we refer to [38] and [39, Sect. 2.6] in linear-quadratic settings, [39, Sect. 4.4] in a crowd-motion example, [67, 68] in SIR models, [69] in wireless networks, and [50] in energy markets to cite a few.

For the purpose of this paper we define them as follows, where the $\inf$ and the $\sup$ are taken over $\boldsymbol{\alpha }^\mathrm{{MFG}}$ in the set of Nash equilibria:
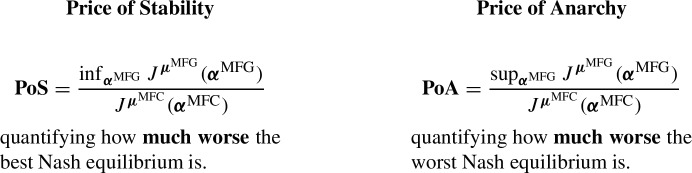


## From MFG to MFC: Incentivization to Reach Social Optimum

In this section, we show how we can incentivize non-cooperative players through modifying the data (i.e., the cost functional) of the MFG model either to make them *attain the optimal cost* or *adopt the optimal behavior* of the original MFC problem, even though they still behave in a non-cooperative manner and reach an MFG Nash equilibrium for the modified data. As emphasized earlier, the results of Sect. 3 are reminiscent of *mechanism design*. Indeed, we show that we can incentivize players to behave in a prescribed way. However, it is not done by designing an objective for a *regulator* or by offering a *contract* to the players. Instead, we modify the data of the existing MFG model in such a way that non-cooperative players reaching an MFG Nash equilibrium in the perturbed model would attain the same minimal cost as in the original cooperative model, or would behave exactly as they would have done in the original cooperative model.

### Matching the MFC Optimal Value

In this subsection, we analyze how to coerce non-cooperative players (by modification of their cost functions) into an equilibrium behavior leading to the optimal cost of the social planner’s original optimization. Since our goal is to match the value of the optimum we use the value functions of the MFC and MFG models to identify the needed perturbation of the model’s cost functions.

We first recall the definition of the value function of the MFC problem, see Definition 2 and (10). Let $\boldsymbol{X}^{\mathrm{{MFC}}, t, \mu }$ be the solution of the following optimization problem: for any $t \in [0,T]$ and $\xi \in L^2(\Omega ,{\mathcal {F}}_{t},{\mathbb {P}};{\mathbb {R}}^d)$ if we denote by $\mu ={\mathcal {L}}(\xi )$ the law of $\xi$, we define the value function $v(t,\mu )$ as

17 $$\begin{aligned} v(t,\mu )=\inf _{\boldsymbol{\alpha }_{\vert [t,T]}} {\mathbb {E}}\left[ \int _t^{T} f\left( s,X_s^{\boldsymbol{\alpha }}, {{\mathcal {L}}}(X_{s}^{\boldsymbol{\alpha }}),\alpha _{s}\right) ds +g\left( X_{T}^{\boldsymbol{\alpha }},{{\mathcal {L}}}(X_{T}^{\boldsymbol{\alpha }} ) \right) \right] , \end{aligned}$$

where the state process $\boldsymbol{X}^{\boldsymbol{\alpha }, t, \mu }$ satisfies the state dynamics (2) on [*t*, *T*] with initial condition $X_{t}^{\boldsymbol{\alpha }} = \xi$. We recall that *f* is defined in (4) to simplify the presentation. Here, the infimum is taken over ${\mathbb {R}}^d$-valued square-integrable $({\mathcal {F}}_{s})_{s \in [t,T]}$-progressively measurable processes $\boldsymbol{\alpha }_{\vert [t,T]} =(\alpha _{s})_{ s \in [t,T]}$. For existence results, see e.g., [63, Theorems 2.2 and 2.3]. Next, we define the extended value function $(t,x,\mu )\mapsto V(t,x,\mu )$ by conditioning on the initial state of the controlled diffusion, namely, for each $(t,x,\mu )\in [0,T]\times {\mathbb {R}}^d\times {{\mathcal {P}}}_{2}({\mathbb {R}}^d)$,

18 $$\begin{aligned} \begin{aligned} V(t,x,\mu )&= {\mathbb {E}}\bigg [ \int _t^T f\bigl (s,X_s^{\mathrm{{MFC}}, t, \mu },{\mathcal {L}}(X_s^{\mathrm{{MFC}}, t, \mu }),{\alpha }^{\mathrm{{MFC}}, t, \mu }_s\bigr )ds\\&\quad +g\bigl (X_T^{\mathrm{{MFC}}, t, \mu },{\mathcal {L}}(X_T^{\mathrm{{MFC}}, t, \mu })\bigr )\, \big | \, X_{t}^{\mathrm{{MFC}}, t, \mu } = x\bigg ], \end{aligned} \end{aligned}$$

where $\boldsymbol{\alpha }^{\mathrm{{MFC}}, t, \mu }$ is the minimizer (which is unique under Assumption 3) in (17) under the constraint ${\mathcal {L}}(X^{\mathrm{{MFC}}, t, \mu }_t)=\mu$. The extended value function *V* satisfies [15, Definition 6.30]

19 $$\begin{aligned} v(t,\mu ) = \int _{{\mathbb {R}}^d} V(t,x,\mu ) d\mu (x). \end{aligned}$$

Our goal is to identify *V*, when it is smooth, as the value function of an MFG with modified cost functions. As we just highlighted, this implicitly requires the minimizer to be unique, as multiplicity of the minimizers are associated with singularities of the value function. In order to proceed with the identification of *V*, we introduce the following modified cost function:

20 $$\begin{aligned} {\tilde{f}}(t,x,\mu ,\alpha )=\frac{1}{2} |\alpha |^2+{\tilde{f}}_0(t,x,\mu ), \end{aligned}$$

where, using a dot $\cdot$ to denote the inner product in ${\mathbb {R}}^d$,

21 $$\begin{aligned} \begin{aligned} {\tilde{f}}_0(t,x,\mu )&= f_0(x,\mu ) +\frac{1}{2} \int _{{\mathbb {R}}^d}\int _{{\mathbb {R}}^d} \partial _{\mu } V(t,x',\mu )(x)\cdot \partial _{\mu } V(t,x'',\mu )(x) d\mu (x')d\mu (x'') \\&\quad -\int _{{\mathbb {R}}^d} \int _{{\mathbb {R}}^d} \partial _{\mu } V(t,x',\mu )({\tilde{x}}) \cdot \partial _{\mu } V(t,x,\mu )({\tilde{x}}) d\mu (x')d\mu ({\tilde{x}}). \end{aligned} \end{aligned}$$

#### Theorem 1

Assume that *V* is continuous, once differentiable in *t*, twice differentiable in *x* with continuous derivatives in $(t,x,\mu )$, the derivative $\partial _x V$ being bounded. Assume further that *V* is differentiable in $\mu$ and that its derivative $(t,x,\mu ,x') \mapsto \partial _\mu V(t,x,\mu )(x')$ is jointly continuous and bounded, and partially differentiable in $x'$ with the derivative $\partial _{x'}\partial _\mu V$ being continuous and bounded. Then, for any initial distribution, the MFG problem with costs $({\tilde{f}}, g)$ has an equilibrium control ${\boldsymbol{\alpha }}^\mathrm{{MFG}}$ that shares the same value function (18) as the minimizer of the MFC problem with costs (*f*, *g*), namely, for any $t\in [0,T]$ and $x \in {{\mathbb {R}}}^d$,

$$\begin{aligned}&{\mathbb {E}}\left[ \int _t^T {{\tilde{f}}}\left( s,X_s^\mathrm{{MFG}},{\mathcal {L}}(X_s^\mathrm{{MFG}}),{\alpha }^\mathrm{{MFG}}_s\right) ds +g\left( X_T^\mathrm{{MFG}},{\mathcal {L}}(X_T^\mathrm{{MFG}})\right) \, \big | \, X_{t}^\mathrm{{MFG}} = x\right] \\&\quad = V\left( t,x,{\mathcal {L}}(X_t^{{\boldsymbol{\alpha }}^\mathrm{{MFC}}})\right) . \end{aligned}$$

For further discussion on the smoothness of *V*, please refer to Appendix 1.

#### Proof of Theorem 1

Remember that the extended value function for the MFC problem is given by (18). In fact, it turns out that the MFC optimizer is given as $\alpha ^{\mathrm{{MFC}}}_s=\bar{\alpha }(s,X^{{\hat{\boldsymbol{\alpha }}}}_s,{\mathcal {L}}(X^{{\hat{\boldsymbol{\alpha }}}}_s))$ for some deterministic function $(t,x,\mu ) \mapsto \bar{\alpha }(t,x,\mu )$ which is given by:

22 $$\begin{aligned} \begin{aligned} \bar{\alpha }(t,x,\mu )&= \hat{\alpha }\left( t,x,\mu ,\partial _{x} V(t,x,\mu ) + \int _{{\mathbb {R}}^d} \partial _{\mu } V(t,x',\mu )(x) d\mu (x')\right) \\&= -\partial _{x} V(t,x,\mu ) - \int _{{\mathbb {R}}^d} \partial _{\mu } V(t,x',\mu )(x) d\mu (x'), \end{aligned} \end{aligned}$$

where we recall that ${\hat{\alpha }}$ is defined in (6), and where the extended value function solves the following master equation:

23 $$\begin{aligned} \begin{aligned}&\partial _{t} V(t,x,\mu ) + \bar{\alpha }(t,x,\mu ) \partial _{x} V(t,x,\mu ) \\&\hspace{15pt} + \frac{\sigma ^2}{2} \Delta V(t,x,\mu ) +\frac{1}{2} |\bar{\alpha }(t,x,\mu )|^2+f_0(x,\mu ) \\&\hspace{15pt} +\int _{{\mathbb {R}}^d} \biggl [ \bar{\alpha }(t,x',\mu ) \partial _{\mu } V(t,x,\mu )(x') + \frac{\sigma ^2}{2}\textrm{trace} \Bigl ( \partial _{x'} \partial _{\mu } V(t,x,\mu )(x') \Bigr ) \biggr ]d\mu (x') = 0, \end{aligned} \end{aligned}$$

for $(t,x,\mu ) \in [0,T] \times {\mathbb {R}}^d \times {\mathcal {P}}_{2}({\mathbb {R}}^d)$, with the terminal condition $V(T,x,\mu ) = g(x,\mu )$. The interested reader can refer to [15, Chapter 6] and [62] for further discussion.

Plugging the relationship (22) into (23), we obtain the full-fledged form of the master equation:

24 $$\begin{aligned} \begin{aligned}&\partial _{t} V(t,x,\mu ) + \frac{\sigma ^2}{2} \Delta V(t,x,\mu ) - \frac{1}{2} |\partial _x V(t,x,\mu ) |^2 \\&\hspace{5pt} +\frac{1}{2} \int _{{\mathbb {R}}^d}\int _{{\mathbb {R}}^d} \partial _{\mu } V(t,x',\mu )(x)\partial _{\mu } V(t,x'',\mu )(x) d\mu (x')d\mu (x'') +f_0(x,\mu ) \\&\hspace{5pt} +\int _{{\mathbb {R}}^d} \biggl [\biggl ( -\partial _x V(t,{\tilde{x}},\mu ) - \int _{{\mathbb {R}}^d} \partial _{\mu } V(t,x',\mu )({\tilde{x}}) d\mu (x') \biggr ) \cdot \partial _{\mu } V(t,x,\mu )({\tilde{x}}) \\&\hspace{5pt} +\frac{\sigma ^2}{2}\textrm{trace} \Bigl (\partial _{\tilde{x}} \partial _{\mu } V(t,x,\mu )({\tilde{x}}) \Bigr ) \biggr ]d\mu ({\tilde{x}}) = 0, \end{aligned} \end{aligned}$$

for $(t,x,\mu ) \in [0,T] \times {\mathbb {R}}^d \times {\mathcal {P}}_{2}({\mathbb {R}}^d)$, with the terminal condition $V(T,x,\mu ) = g(x,\mu )$.

Now, our goal is to identify *V* as the value function of an MFG with perturbed cost functions. We do this by identifying the above master equation as the master equation of an MFG problem with a different cost function. In order to do so, we use the fact that the master equation of an MFG with controlled state equation (2) and running cost function ${\tilde{f}}$ defined in (20) is given by:

25 $$\begin{aligned} \begin{aligned}&\partial _{t} U(t,x,\mu ) + \frac{\sigma ^2}{2} \Delta U(t,x,\mu ) - \frac{1}{2}|\partial _{x} U(t,x,\mu )|^2\\&\hspace{5pt} - \int _{{\mathbb {R}}^d} \partial _{x} {U}(t,x',\mu ) \cdot \partial _{\mu } U(t,x,\mu )(x') d\mu (x')\\&\hspace{5pt} + \frac{\sigma ^2}{2} \int _{{\mathbb {R}}^d} \text { trace } \Bigl [ \partial _{x'} \partial _{\mu } U(t,x,\mu ) (x') \Bigr ] d\mu (x') + {\tilde{f}}_0(t,x,\mu ) = 0, \end{aligned} \end{aligned}$$

with terminal condition $U(T,x,\mu )=g(x,\mu )$. See e.g., [17, 62] for more details on the derivation of the master equation. By inspection, one sees that the choice of $\tilde{f}_0$ given by (21) does the trick in the sense that it turns the master equation (24) of the MFC into the master equation of an MFG. So if the individual players are incentivized and face the running costs ${\tilde{f}}$ as given by formulas (20) and (21), then in an MFG equilibrium, they will have the same cost as if they were implementing the optimal control of a social planner using the running cost function (4). Here, such an MFG equilibrium does exist, by construction *V* is a classical solution to (24) and this induces a Nash equilibrium by a verification argument, see [15, Proposition 5.108].

#### Remark 2

One can design new MFG models, for instance, one could just consider ${\tilde{f}}_0=0$ and ${\tilde{g}}=J^{*}$ (i.e., ${\tilde{g}}$ is a constant cost, equal to the optimal cost in the MFC problem introduced in (10)). The optimizer in the resulting MFG is zero and the equilibrium cost is obviously equal to $J^{*}$. The very interest of our design is that not only the optimal costs are the same, but also the conditional remaining costs of the individuals are the same in the two approaches. In other words, the value function $V(t,x,\mu )$ at time *t* when the player is in state *x* and the measure is in state $\mu$ is the same in the newly designed MFG and the original MFC, which is a very much stronger result. We emphasize that this would not hold in the trivial case when ${\tilde{f}}_0=0$ and ${\tilde{g}}=J^{*}$. Accordingly, we say that our incentivization design is Markovian.

#### Remark 3

Notice that in order to implement the incentives discussed in Theorem 1, one needs to compute the value function *V* (or at least its derivative $\partial _\mu V$). Besides linear-quadratic models, in general there is no closed-form solution for *V* and numerical methods should be used. For instance, [70] proposed a deep learning method based on symmetric neural networks and [71, 72] proposed reinforcement learning methods to compute the MFC value function.

We want to emphasize that although in the new MFG, the value for a representative player is the same as the MFC optimum of the original model, the players’ dynamics are not the same in general. Indeed, remember that the players’ dynamics are directly driven by the control, see (2). In the MFC with running cost *f*, the social optimum is attained by using the control $\bar{\alpha }$ defined in (22): $\alpha ^{\mathrm{{MFC}}}_s=\bar{\alpha }(s,X^{\mathrm{{MFC}}}_s,{\mathcal {L}}(X^{\mathrm{{MFC}}}_s))$. In the new MFG with running cost $\tilde{f}$ defined in (20), the MFG equilibrium control is obtained by using the control given by the minimizer of the Hamiltonian:

$$\begin{aligned} \tilde{H}(t,x,\mu ,y,\alpha ) =\alpha y + \frac{1}{2} |\alpha |^2 + \tilde{f}_0(x,\mu ) \end{aligned}$$

along the solution of the MFG. In this situation, the equilibrium control is given by:

$$ \alpha ^\mathrm{{MFG}}_s = - \partial _x U\left( s,X^\mathrm{{MFG}}_s,{\mathcal {L}}\left( X^\mathrm{{MFG}}_s\right) \right) , $$

where we recall that *U* is the solution of the master equation defined in (25). Now, for the sake of contradiction, assume that $\bar{\boldsymbol{\alpha }} = \boldsymbol{\alpha }^\mathrm{{MFG}}$. Then ${\mathcal {L}}(X^\mathrm{{MFC}}_s) = {\mathcal {L}}(X^\mathrm{{MFG}}_s)$. Using (6) and the fact that $V = U$, we deduce that the equality of the two controls implies

$$ \int _{{\mathbb {R}}^d} \partial _{\mu } V(t,x',\mu _s)(x) d\mu _s(x') = 0, $$

where $\mu _s = {\mathcal {L}}(X^\mathrm{{MFC}}_s)$. This is not true in general, hence a contradiction. For an example where this term is non-zero, see [17, Sect. 4.5.1, pp. 311–313].

### Matching the MFC Optimal Behavior

We now show that one can coerce non-cooperative players in an MFG (by modifying their running and terminal cost functions) into *behaving* exactly as if they were adopting the MFC optimal control identified by a social planner optimizing the original MFC cost.

#### Incentivization Mechanism in MFG

We identify the MFG equilibrium via the FBSDE of MKV type derived from the stochastic Pontryagin principle. The starting point is the characterization of the behavior (in terms of control and state processes) of individual players adopting the prescriptions identified by the social planner’s optimization. As explained in Sect. 2.4, the state dynamics at the MFC optimum are given by the forward component of the solution of the FBSDE (15) while the MFC optimal control is given by $\alpha ^{\mathrm{{MFC}}}_t=-Y_t$ for $t\in [0,T]$.

Now, we introduce new running and terminal cost functions for the MFG defined in Sect. 2.3 with the running cost (4). For each $\lambda \in [0,1]$, we define a new cost function $f_\lambda$ by:

26 $$\begin{aligned} f_\lambda (x,\mu )= f_0(x,\mu ) +\lambda \tilde{\mathbb {E}}\left[ \frac{\delta f_0}{\delta m}({\tilde{X}},\mu )(x)\right] , \quad (x,\mu ) \in {{\mathbb {R}}}^d \times {{\mathcal {P}}}_2({{\mathbb {R}}}^d), \end{aligned}$$

where $\tilde{X}$ has distribution $\mu$, recalling the notations introduced in Sect. 2.1.

Now, we consider the MFG with controlled state dynamics (2), a running cost function:

$$ \frac{1}{2}|\alpha |^2+f_\lambda (x,\mu ), $$

and a terminal cost function $g_\lambda (x,\mu )= g(x,\mu ) +\lambda \tilde{\mathbb {E}}[\frac{\delta g}{\delta m}({\tilde{X}},\mu )(x)]$.

##### Theorem 2

Assume that the system (15) is uniquely solvable and that $f_1$ and $g_1$ (together with $\sigma$) satisfy Assumption 2. Then, for $\lambda =1$, the MFG associated with $(f_1,g_1)$ has the same solution as the original MFC problem (Definition 2).

Before presenting the proof, let us recall that, under Assumption 3, the system (15) is uniquely solvable. As far as $f_1$ and $g_1$ are concerned, Assumption 2.(*i*) is satisfied if $f_0$ and *g* are Lipschitz continuous in $(x,\mu )$ when ${{\mathcal {P}}}_2({{\mathbb {R}}}^d)$ is equipped with the Total Variation distance. This forces the derivatives $\delta f_0/\delta m$ and $\delta g_0/\delta m$ to be bounded. The Assumption 2.(*ii*) holds when $f_0$ and *g* satisfy the forms in Remark 1 with $\varphi _0$ and $\psi$ convex.

##### Proof of Theorem 2

We first start by identifying equilibria in the MFG driven by $(f_\lambda , g_\lambda )$ for $\lambda \in [0,1]$ via the stochastic maximum principle with the new cost functions. The MFG equilibrium state dynamics are given by the forward component of the solution of the FBSDE:

27 $$\begin{aligned} {\left\{ \begin{array}{ll} dX_t& = -Y_tdt +\sigma dW_t\\ dY_t& =-\partial _x f_\lambda (X_t,{\mathcal {L}}(X_t)) dt + Z_t dW_t,\\ Y_T& =\partial _x g_\lambda (X_T,{\mathcal {L}}(X_T)), \end{array}\right. } \end{aligned}$$

while since the dependence of the Hamiltonian with respect to control is the same as before, the equilibrium control is still given by $\alpha _t=-Y_t$ for $t\in [0,T]$. Clearly, this MFG equilibrium coincides with our original MFG equilibrium as characterized by (7) when $\lambda =0$. Moreover, since the L-derivative and the functional derivatives are related by the formula (1) (with $U = f$ here), we see that the solution of this MFG as given by (27) coincides for $\lambda =1$ with the solution of the FBSDE (15), which is known, under the standing assumption, to be the unique MFC minimizer.

Now, it remains to observe from the additional assumption we made on $(f_1,g_1)$ that, for any equilibrium $(\mu _t)_{t\in [0,T]}$ of the MFG driven by $(f_1,g_1)$, the cost functional in the environment $(\mu _t)_{t\in [0,T]}$ has a unique minimizer, which is uniquely characterized by the stochastic maximum principle, see for instance [15, Chapter 3]. Therefore, there is a one-to-one mapping between the equilibria of the MFG driven by $(f_1,g_1)$ and the solutions to (15).

##### Remark 4

The interpretation of the statement is as follows: Without the intervention of the social planner and without forcing all the players to use the control identified by someone else, we can get to the same state trajectory and behavior by letting the individual players settle in a Nash equilibrium for an MFG with perturbed cost functions where the perturbations can be interpreted as incentives. Furthermore, when $\lambda =1$, the MFG is a potential game in the sense that MFG equilibrium is equivalent to an MFC solution. Furthermore, we stress that the intervention of Theorem 2 simply requires computing derivatives of the cost functions $f_0$ and *g*. It does not require computing the value function, as was the case in the previous subsection for Theorem 1.

#### Another Interpretation: $\lambda$-Interpolated MFGs

The procedure introduced in the above subsection provides an interpolation (parameterized by $\lambda$) between the solution(s) of a given MFG and the solution(s) of a given MFC with the same state equation and running and terminal cost functions. Our goal in this section is to study this interpolation. We will start by giving another interpretation of the FBSDE system (27).

We introduce, for a generic flow ${\boldsymbol{\mu }}:=(\mu _t)_{t\in [0,T]}$ the cost $J^{\lambda ,\textrm{MF}} ( \boldsymbol{\alpha }; {\boldsymbol{\mu }} )$:

28 $$\begin{aligned} \begin{aligned} J^{\lambda ,\textrm{MF}} \bigl ( \boldsymbol{\alpha }; {\boldsymbol{\mu }} \bigr )&:= (1-\lambda ) J\bigl ( {\boldsymbol{\alpha }};{\boldsymbol{\mu }}\bigr ) + \lambda J^{\mathrm{{MFC}}} \bigl ( {\boldsymbol{\alpha }}\bigr ) \\&= {{\mathbb {E}}} \biggl [ \frac{1}{2} \int _0^T \vert \alpha _t \vert ^2 dt + \int _0^T \Bigl [ (1-\lambda ) f_0 \bigl (X_t^{\boldsymbol{\alpha }},\mu _t\bigr ) + \lambda f_0 \bigl ( X_t^{\boldsymbol{\alpha }},{{\mathcal {L}}}(X_t^{\boldsymbol{\alpha }}) \bigr ) \Bigr ] dt \biggr ] \\&\quad + {{\mathbb {E}}} \Bigl [ (1-\lambda ) g \bigl ( X_T^{\boldsymbol{\alpha }}, \mu _T \bigr ) + \lambda g \bigl ( X_T^{\boldsymbol{\alpha }}, {{\mathcal {L}}}(X_T^{\boldsymbol{\alpha }}) \bigr ) \Bigr ]. \end{aligned} \end{aligned}$$

##### Definition 3

For a given $\lambda \in [0,1]$, we say that a (square-integrable) control $\boldsymbol{\alpha }^{\boldsymbol{\lambda }}$ induces a $\lambda$-interpolated mean field (MF) equilibrium if $\boldsymbol{\alpha }^{\boldsymbol{\lambda }}$ solves the minimization problem

$$\begin{aligned} \inf _{\boldsymbol{\alpha }} J^{\lambda ,\textrm{MF}} \bigl ( \boldsymbol{\alpha }; {\boldsymbol{\mu }}^{\lambda } \bigr ), \end{aligned}$$

where ${\boldsymbol{\mu }}^{\lambda }:= (\mu _t^{\lambda })_{t\in [0,T]}$ with $\mu _t^{\lambda } = {{\mathcal {L}}}(X_t^{\lambda })$ for $t \in [0,T]$ where $\boldsymbol{X}^{\lambda }$ solves (2) controlled by $\boldsymbol{\alpha }^{\lambda }$.

We emphasize that 0-interpolated MF equilibrium is an MFG equilibrium and a 1-interpolated MF equilibrium is an MFC optimum. This problem falls in the category of *mean field control games* introduced in [73, 74]. Such games are an extension of MFGs where, given the population distribution flow, each player solves an MFC problem instead of a standard stochastic control problem. In this case too, the cost depends on the population distribution as well as on the individual player’s distribution. In fact, we can write:

$$ J^{\lambda ,\textrm{MF}}\bigl ( \boldsymbol{\alpha }; {\boldsymbol{\mu }} \bigr ):= {{\mathbb {E}}} \biggl [ \frac{1}{2} \int _0^T \vert \alpha _t \vert ^2 dt + \int _0^T \tilde{f}_{\lambda } \bigl (X_t^{\boldsymbol{\alpha }}, \mu _t, {{\mathcal {L}}}(X_t^{\boldsymbol{\alpha }}) \bigr ) dt + \tilde{g}_\lambda \bigl ( X_T^{\boldsymbol{\alpha }}, \mu _T, {{\mathcal {L}}}(X_T^{\boldsymbol{\alpha }})\bigr )\biggr ], $$

with $\tilde{f}_\lambda (x, \mu , \tilde{\mu }) = (1-\lambda ) f_0 \bigl (x,\mu \bigr ) + \lambda f_0 \bigl ( x, \tilde{\mu }\bigr )$ and $\tilde{g}_\lambda (x, \mu , \tilde{\mu }) = (1-\lambda ) g \bigl (x,\mu \bigr ) + \lambda g \bigl ( x, \tilde{\mu }\bigr )$, where $\mu$ and $\tilde{\mu }$ play the role of the distributions called respectively *global* and *local* in [73, 74].

##### Proposition 3

Let $\lambda \in [0,1]$. Assume that $\boldsymbol{\alpha }^{\lambda }$ is a $\lambda$-interpolated mean field equilibrium. Then, the pair of processes $(X_t,Y_t)_{0 \le t \le T}=(X_t^{\lambda },-\alpha ^{\lambda }_t)_{0 \le t \le T}$ solves the system (27).

##### Proof of Proposition 3

The proof is implemented by utilizing the stochastic Pontryagin maximum principle for controlled MKV processes. When the environment $\boldsymbol{\mu }$ is fixed, the minimizers of $J^{\lambda ,\textrm{MF}}(\cdot \,; {\boldsymbol{\mu }})$ solves the system (27) but with $f_\lambda (X_t, {{\mathcal {L}}}(X_t))$ replaced by $(1-\lambda ) f_0(X_t,\mu _t)+ \lambda f_0(X_t,{{\mathcal {L}}}(X_t))$ and similarly for $g_\lambda$. Under the fixed point condition ${\boldsymbol{\mu }}=({{\mathcal {L}}}(X_t))_{t\in [0,T]}$, we get (27).

Intuitively, this interpolation can be viewed as a situation in which an *invisible hand* tunes the cost function: starting from the MFG setting, the cost gradually incorporates more and more of the MFC setting. We can imagine that this process is done iteratively: at each step, the invisible hand changes the previous cost (by increasing $\lambda$), making it a bit more similar to the MFC cost; then the players compute a Nash equilibrium for this modified game, before moving to the next step.

#### Continuous Deformation from MFG to MFC

An interesting question in practice is whether we can find, for each $\lambda \in [0,1]$, a $\lambda$-interpolated mean field equilibrium such that the resulting equilibria are continuous w.r.t. the parameter $\lambda$: intuitively, this would mean that there exists a continuous deformation connecting an MFG equilibrium and an MFC optimum. In order words, for every $\lambda \in [0,1]$, the trajectory of a representative player in the Nash equilibrium changes smoothly when $\lambda$ changes. We can easily provide some intuitive applications: when playing games repeatedly, this would allow to drive continuously the equilibrium state of the population to a social optimum. Continuity implies that there is no big jump in the successive costs paid by the players during these iterations. From a modeling point of view, the invisible hand may want to change the rules in a soft manner that does not perturb the collectivity too abruptly. This question is however difficult to solve in full generality. Indeed, without any extra structural conditions guaranteeing uniqueness, existing compactness methods used in MFG theory to prove existence of an equilibrium are certainly not sufficient to prove the existence of such a continuous deformation. For this reason, we consider specific models for which continuous deformations can be shown to exist by simpler arguments. We have, in line with Remark 1, the following statement.

##### Theorem 4

Assume that $f_0$ and *g* are given as

$$\begin{aligned} f_0(x,\mu ) = \int _{{{\mathbb {R}}}^d} \varphi _0(x-y) d\mu (y), \quad g(x,\mu ) = \int _{{{\mathbb {R}}}^d} \psi (x-y) d\mu (y), \end{aligned}$$

where $\varphi _0$ and $\psi$ are even convex functions on ${{\mathbb {R}}}^d$ with Lipschitz continuous derivatives. Then, for any $\lambda \in [0,1]$, there exists a unique $\lambda$-interpolated mean field equilibrium control $\boldsymbol{\alpha }^\lambda$ and the mapping $[0,1] \ni \lambda \mapsto (X_t^{\lambda })_{t\in [0,T]}$ is continuous when distances between two different values of the right-hand side are understood for the norm $\Vert {{\boldsymbol{X}}} \Vert _{{{\mathbb {S}}}_2}:= \sup _{t\in [0,T]} {{\mathbb {E}}}[ \vert X_t \vert ^2]^{1/2}$.

##### Proof of Theorem 4

In this setting, we even have more than what is stated in the statement. Recalling Sect. 2.1, we can observe that:

$$\begin{aligned} \tilde{{\mathbb {E}}} \bigl [ \frac{\delta f_0}{\delta m}\bigl ( {\tilde{X}},\mu \bigr )(x)\bigr ]= {{\mathbb {E}}} \bigl [ \varphi _0 \bigl ( X-x) \bigr ] = f_0(x,\mu ), \end{aligned}$$

where *X* is a random variable with $\mu$ as its distribution. Following (26), $f_\lambda (x,\mu )=(1+\lambda ) f_0(x,\mu )$ and the system (27) is not only the mean field forward-backward system that arises when applying the Pontryagin principle to describe the MFG equilibria driven by the two costs $f_\lambda$ and $g_\lambda$, but it is also the Pontryagin system deriving from the MFC problem associated with the running cost

$$\begin{aligned} \mu \mapsto \frac{1+\lambda }{2} \int _{{{\mathbb {R}}}^d} \int _{{{\mathbb {R}}}^d} \varphi _0(x-y) d \mu (x) d \mu (y), \end{aligned}$$

and the terminal cost

$$\begin{aligned} \mu \mapsto \frac{1+\lambda }{2} \int _{{{\mathbb {R}}}^d} \int _{{{\mathbb {R}}}^d} \psi (x-y) d \mu (x) d\mu (y). \end{aligned}$$

Following Assumptions 1 and 3 (see also Remark 1), this MFC problem has a unique solution, which is fully characterized by the maximum principle. Hence there exists at most one $\lambda$-interpolated MF equilibrium. As for existence, the point is to check that the Pontryagin system (27) is, in this setting, a sufficient condition. This follows from the convexity conditions on the coefficients, which imply that Assumption 2 is in force where the properties of $f_\lambda$ and $g_\lambda$ are deduced from the ones of $f_0$ and *g*. As for the continuity w.r.t. $\lambda$, we take advantage of the convex structure of the coefficients in order to bypass any compactness arguments, which would be more technical. Indeed, the functions in the system (27) satisfy the following monotonicity condition:

29 $$\begin{aligned} \begin{aligned} {{\mathbb {E}}} \Bigl [ \Bigl \langle \partial _x f_\lambda \bigl ( X, {{\mathcal {L}}}(X)\bigr ) - \partial _x f_\lambda \bigl ( X',{{\mathcal {L}}}(X')\bigr ), X-X' \Bigr \rangle \Bigr ] \ge 0, \end{aligned} \end{aligned}$$

for any two random variables $X,X' \in L^2(\Omega ,{{\mathcal {A}}},{{\mathbb {P}}};{{\mathbb {R}}}^d)$. The point is then to prove stability by expanding the inner product ${{\mathbb {E}}} \bigl [ \bigl \langle X_t^\lambda - X_t^{\lambda '}, Y_t^\lambda - Y_t^{\lambda '} \bigr \rangle \bigr ].$ By using (27), we have

$$\begin{aligned} \begin{aligned}&\frac{d}{dt} {{\mathbb {E}}} \bigl [ \bigl \langle X_t^\lambda - X_t^{\lambda '}, Y_t^\lambda - Y_t^{\lambda '} \bigr \rangle \bigr ] \\&\quad = - {{\mathbb {E}}} \Bigl [ \bigl \vert Y_t^\lambda - Y_t^{\lambda '} \bigr \vert ^2\Bigr ] - {{\mathbb {E}}} \Bigl [ \Bigl \langle \partial _x f_{\lambda '}\bigl ( X_t^\lambda , {{\mathcal {L}}}(X_t^{\lambda })\bigr ) - \partial _x f_{\lambda '} \bigl ( X_t^{\lambda '},{{\mathcal {L}}}(X_t^{\lambda '})\bigr ), X_t^\lambda -X_t^{\lambda '} \Bigr \rangle \Bigr ] \\&\qquad - {{\mathbb {E}}} \Bigl [ \Bigl \langle \partial _x f_{\lambda }\bigl ( X_t^\lambda , {{\mathcal {L}}}(X_t^{\lambda })\bigr ) - \partial _x f_{\lambda '} \bigl ( X_t^{\lambda },{{\mathcal {L}}}(X_t^{\lambda })\bigr ), X_t^\lambda -X_t^{\lambda '} \Bigr \rangle \Bigr ], \end{aligned} \end{aligned}$$

and then, expressing $\partial _x f_{\lambda } - \partial _x f_{\lambda '}$ in terms of $(\lambda -\lambda ')$ by using their Lipschitz continuity and performing a similar expansion for the boundary condition, we get by using (29):

$$\begin{aligned} \begin{aligned} {{\mathbb {E}}} \int _0^T \vert Y_t^{\lambda } - Y_t^{\lambda '} \vert ^2 dt \le C_\lambda \vert \lambda - \lambda ' \vert \sup _{0 \le t \le T} {{\mathbb {E}}}\bigl [ \vert X_t^{\lambda } - X_t^{\lambda '} \vert ^2 \bigr ]^{1/2}, \end{aligned} \end{aligned}$$

where the constant $C_\lambda$ depends on the second order moments of $(X_t^\lambda )_{t\in [0,T]}$. Using the fact that $|X_t^{\lambda } - X_t^{\lambda '}| \le \int _0^t|Y_s^{\lambda } - Y_s^{\lambda '}|ds$ and plugging this in the right-hand side above, we deduce that

$$\begin{aligned} \begin{aligned} {{\mathbb {E}}} \int _0^T \vert Y_t^{\lambda } - Y_t^{\lambda '} \vert ^2 dt \le C_\lambda \vert \lambda - \lambda ' \vert ^2, \end{aligned} \end{aligned}$$

for a possibly new value of $C_\lambda$ and then

$$\begin{aligned} \sup _{0 \le t \le T} {{\mathbb {E}}}\bigl [ \vert X_t^{\lambda } - X_t^{\lambda '} \vert ^2 \bigr ]^{1/2} \le C_\lambda \vert \lambda - \lambda ' \vert , \end{aligned}$$

which completes the proof.

##### Example 1

We provide two examples, with $x\in {\mathbb {R}}$ and $\mu \in \mathcal {P}({\mathbb {R}})$, where the assumptions of Theorem 4 holds:

(i) $f_0(x,\mu )=g(x, \mu ) = \int _{\mathbb {R}}(x-y)^2d\mu (y).$ We emphasize that this form satisfies Assumption 1, since $f_0$ and *g* are continuously differentiable in both arguments and the derivatives are jointly Lipschitz continuous, realizing we have $\partial _x f_0(x,\mu ) = 2x-2\int _{{\mathbb {R}}}y d\mu (y)$ and $\partial _\mu f_0(x,\mu )(X) =2X-2x$ (similarly for *g*).
(ii) $f_0(x,\mu )=g(x, \mu ) = \int _{\mathbb {R}}\sqrt{1+|x-y|^2}d\mu (y)$. We emphasize that this form satisfies Assumption 1, since $f_0$ and *g* are continuously differentiable in both arguments and the derivatives are jointly Lipschitz continuous. Furthermore, it also satisfies Assumption 2.(ii) since they are convex in *x* and $x \partial _x f_0(0, \delta _x) = x (-\partial _x \sqrt{1+|x|^2}) = - \frac{|x|^2}{\sqrt{1+|x|^2}}$ that behaves linearly at infinity. Therefore the condition holds for $C\ge 1$.

#### The Case of Monotone Interactions

We now address the case when the two cost functions $f_0$ and *g* satisfy the Lasry–Lions monotonicity condition:

30 $$\begin{aligned} \forall m, m' \in {{\mathcal {P}}}({{\mathbb {R}}}^d), \quad \int _{{{\mathbb {R}}}^d} \Bigl ( f_0(x,m') - f_0(x,m) \Bigr ) d \bigl ( m' - m \bigr ) (x) \ge 0, \end{aligned}$$

and similarly for *g*.

##### Example 2

We provide an example with $x\in {\mathbb {R}}, \mu \in \mathcal {P}({\mathbb {R}})$ where this condition holds:

$$\begin{aligned} f_0(x,\mu )=\frac{c_1}{2}x^2,\ g(x,\mu )=\frac{c_2}{2}x^2+ \frac{c_3}{2} \left( \int _{\mathbb {R}}y d\mu (y)\right) ^2. \end{aligned}$$

We emphasize that Assumption 1 holds, since the functions are continuously differentiable in both arguments and the derivatives are jointly Lipschitz continuous, realizing $\partial _\mu f_0(x,\mu )(X)=0$ and $\partial _\mu g(x,\mu )(X) = c_3{\mathbb {E}}_\mu [X]$. Furthermore, Assumption 2.(ii) also holds since they are convex in *x* and $x\partial _x f_0(0, \delta _x)=0>-C(1+|x|)$ holds for any $C>0$ (similarly for *g*).

Under Lasry–Lions monotonicity condition, we have the following statement:

##### Proposition 5

Let $0 \le \lambda < \lambda ' \le 1$. Assume that ${\boldsymbol{\alpha }}^{\lambda }$ (together with the flow ${\boldsymbol{\mu }}^\lambda$) and ${\boldsymbol{\alpha }}^{\lambda '}$ (together with the flow ${\boldsymbol{\mu }}^{\lambda '}$) are two interpolated MF equilibria, respectively with $\lambda$ and $\lambda '$ as parameters. Then, necessarily

$$\begin{aligned} J^{\lambda ', \mathrm MF}\bigl ( {\boldsymbol{\alpha }}^{\lambda '};{\boldsymbol{\mu }}^{\lambda '} \bigr ) \le J^{\lambda , \mathrm MF}\bigl ( {\boldsymbol{\alpha }}^{\lambda };{\boldsymbol{\mu }}^{\lambda } \bigr ). \end{aligned}$$

Moreover, if $\lambda ' <1$ and $f_0$ or *g* is strictly monotone, then the inequality is strict.

##### Proof of Proposition 5

By optimality of the two equilibria w.r.t. their corresponding parameter, we have the following two inequalities:

$$\begin{aligned} \begin{aligned} J^{\lambda ,\textrm{MF}}\bigl ( \boldsymbol{\alpha }^{\lambda };\boldsymbol{\mu }^{\lambda })&\le J^{\lambda ,\textrm{MF}}\bigl ( \boldsymbol{\alpha }^{\lambda '};\boldsymbol{\mu }^{\lambda }), \\ J^{\lambda ',\textrm{MF}}\bigl ( \boldsymbol{\alpha }^{\lambda '};\boldsymbol{\mu }^{\lambda '})&\le J^{\lambda ',\textrm{MF}}\bigl ( \boldsymbol{\alpha }^{\lambda };\boldsymbol{\mu }^{\lambda '}). \end{aligned} \end{aligned}$$

Therefore, multiplying the first inequality by $1-\lambda '$ and the second one by $1-\lambda$ and then adding the resulting two inequalities, we get

$$\begin{aligned} \begin{aligned} \bigl ( \lambda ' (1-\lambda ) - \lambda (1-\lambda ')\bigr ) J^\mathrm{{MFC}}\bigl ( \boldsymbol{\alpha }^{\lambda '} \bigr )&\le \bigl ( \lambda '(1-\lambda ) - \lambda (1-\lambda ')\bigr ) J^\mathrm{{MFC}}\bigl ( \boldsymbol{\alpha }^{\lambda } \bigr ) \\&\hspace{15pt} + (1-\lambda ') (1-\lambda ) \bigl ( J\bigl ( \boldsymbol{\alpha }^{\lambda };\boldsymbol{\mu }^{\lambda '}) - J\bigl ( \boldsymbol{\alpha }^{\lambda '};\boldsymbol{\mu }^{\lambda '})\bigr ) \\&\hspace{15pt} + (1-\lambda ) (1-\lambda ') \bigl ( J\bigl ( \boldsymbol{\alpha }^{\lambda '};\boldsymbol{\mu }^{\lambda }) - J\bigl ( \boldsymbol{\alpha }^{\lambda };\boldsymbol{\mu }^{\lambda })\bigr ). \end{aligned} \end{aligned}$$

Using monotonicity to show that $J\bigl ( \boldsymbol{\alpha }^{\lambda };\boldsymbol{\mu }^{\lambda '}) - J\bigl ( \boldsymbol{\alpha }^{\lambda '};\boldsymbol{\mu }^{\lambda '}) - ( J\bigl ( \boldsymbol{\alpha }^{\lambda };\boldsymbol{\mu }^{\lambda }) - J\bigl ( \boldsymbol{\alpha }^{\lambda '};\boldsymbol{\mu }^{\lambda }) ) \le 0$, we obtain:

31 $$\begin{aligned} \begin{aligned} \bigl ( \lambda ' - \lambda \bigr ) J^\mathrm{{MFC}}\bigl ( \boldsymbol{\alpha }^{\lambda '} \bigr )&\le \bigl ( \lambda ' - \lambda \bigr ) J^\mathrm{{MFC}}\bigl ( \boldsymbol{\alpha }^{\lambda } \bigr ), \end{aligned} \end{aligned}$$

which can be reformulated as

$$\begin{aligned} J^{\lambda ',\mathrm MF}\bigl ( \boldsymbol{\alpha }^{\lambda '}; {\boldsymbol{\mu }}^{\lambda '} \bigr ) = J^\mathrm{{MFC}}\bigl ( \boldsymbol{\alpha }^{\lambda '} \bigr ) \le J^\mathrm{{MFC}}\bigl ( \boldsymbol{\alpha }^{\lambda } \bigr ) = J^{\lambda ,\mathrm MF}\bigl ( \boldsymbol{\alpha }^{\lambda }; {\boldsymbol{\mu }}^{\lambda }\bigr ). \end{aligned}$$

This proves the first claim. The second claim is shown by noticing that the inequality (31) becomes strict when $\lambda ' <1$ and one of the two functions, $f_0$ or *g*, is strictly monotone.

We draw several conclusions from the previous lemma, in the monotone setting:

1. First, the equilibrium cost is decreasing with respect to the parameter $\lambda$;
2. Second, interpolated MF equilibria are at most unique when $\lambda <1$ and one of the two functions, $f_0$ or *g* satisfies the Lasry-Lions monotonicity condition strictly.

##### Remark 5

As stated in the second observation above when $\lambda =1$ the uniqueness is not guaranteed. Then, in practice the deformation process can assist us with selecting one of the equilibria at $\lambda =1$ by studying the limit of $\lambda$-interpolated MF equilibrium as $\lambda \rightarrow 1$.

## From MFC to MFG: Deviation from Social Optimum

The goal of this section is to discuss what happens when cooperative players are allowed to deviate from the social planner’s prescribed behavior. In contrast with the notion of Nash equilibrium that formalizes the stability of a game, we argue that social optima are generically unstable. We introduce the Price of Instability (PoI) to quantify this feature of the MFC optimum by letting one infinitesimal player deviate from the social optimum. Later in the section, we introduce the notion of *p*-partial mean field equilibrium where a proportion *p* of the population is allowed to deviate. From there, we introduce and analyze a repeated game model describing iterative deviations, with a varying proportion of players deviating from the social optimum.

### Single Player Deviation: Price of Instability of a Social Optimum

*By how much can a player’s expected cost be lowered by deviating unilaterally from the MFC optimal control* $\boldsymbol{\alpha }^\mathrm{{MFC}}$ *identified by the social planner?* As we argued in Sect. 2, the expected cost for representative player in any MFG equilibrium is higher than the expected cost for a representative player when the players all agree to use the control identified by a social planner that is computed via the solution of the MFC problem. Letting players minimize their individual costs, even when they reach a Nash equilibrium, comes at a cost when compared to a centrally coordinated optimization. Various forms of quantification of this difference in cost have been proposed such as PoS and PoA, as we mentioned in the introduction and in Sect. 2. In this section, we argue that while less costly, the MFC optimum is less stable and we quantify this level of instability. Following the MFC optimal control, the individual player’s cost is

32 $$\begin{aligned} J^*&:= J^\mathrm{{MFC}}(\boldsymbol{\alpha }^\mathrm{{MFC}})\nonumber \\&={\mathbb {E}}\left[ \int _0^T \left( \frac{1}{2}|\alpha ^\mathrm{{MFC}}_t|^2+f_0(X^\mathrm{{MFC}}_t,\mu ^\mathrm{{MFC}}_t)\right) dt + g\left( X^\mathrm{{MFC}}_T,\mu ^\mathrm{{MFC}}_T\right) \right] .\nonumber \\ \end{aligned}$$

Alternatively, if allowed to deviate from this control, still evolving in the same environment, the player’s cost can become

33 $$\begin{aligned} {\hat{J}}_0 := J^{\boldsymbol{\mu }^\mathrm{{MFC}}}({\hat{\boldsymbol{\alpha }}}) = {\mathbb {E}}\left[ \int _0^T \left( \frac{1}{2}|{\hat{\alpha _t}}|^2+f_0({\hat{X}}_t,\mu ^\mathrm{{MFC}}_t)\right) dt + g\left( {\hat{X}}_T,\mu ^\mathrm{{MFC}}_T\right) \right] \end{aligned}$$

where $$d{\hat{X}}_t = {\hat{\alpha }_{t}} dt +\sigma dW_t$$ and

34 $$\begin{aligned} \begin{aligned} \hat{\boldsymbol{\alpha }} = \mathop {\mathrm {arg\,min}}\limits _{\boldsymbol{\alpha }}\  &{\mathbb {E}}\left[ \int _0^T \left( \frac{1}{2}|\alpha _t|^2+f_0(X^{\boldsymbol{\alpha }}_t,\mu ^\mathrm{{MFC}}_t)\right) dt + g(X^{\boldsymbol{\alpha }}_T,\mu ^\mathrm{{MFC}}_T)\right] \\&\text {s.t. } dX^{\boldsymbol{\alpha }}_t = \alpha _t dt + \sigma dW_t. \end{aligned} \end{aligned}$$

#### Definition 4

The Price of Instability (PoI) is defined as the quantity:

35 $$\begin{aligned} \textrm{PoI}= J^* - {\hat{J}}_0 \end{aligned}$$

where $J^*$ is the cost of the MFC problem, and ${\hat{J}}_0$ in (33) is the cost associated with the optimal control in the classical control problem in the environment given by the marginal distribution of the optimal state in the MFC problem.

Notice that PoI does not involve calculation of Nash equilibria in the system. Furthermore, we emphasize that the PoI definition does not require uniqueness of MFC optimal control, since by definition corresponding MFC costs for possibly different MFC optimal controls should be the same. By construction, $\text {PoI}\ge 0$, and an interesting question is to understand when the PoI is strictly positive.

#### Remark 6

If $\textrm{PoI}=0$, ${\boldsymbol{\alpha }}^\mathrm{{MFC}}$ is an MFG equilibrium control. In this case, $\textrm{PoS}=1$, and if the MFG equilibrium is unique, then $\textrm{PoA} = 1$ too. However, in principle, it is possible to have PoI arbitrarily close to 0 and PoA arbitrarily large. This would correspond to a situation where the social optimum is *almost stable* under unilateral deviations, but when all the players deviate unilaterally, the situation gradually evolves toward a Nash equilibrium with a much higher expected cost.

We recall from [63, Theorems 2.2, 2.3] that any optimal control can be assumed to be in a feedback form, i.e., $({\alpha }_t^\mathrm{{MFC}})_{t\in [0,T]}= (\alpha (t,X_t^\mathrm{{MFC}}))_{t\in [0,T]}$. We then have the following result:

#### Proposition 6

Assume that ${\boldsymbol{\alpha }}^\mathrm{{MFC}}$ can be written in a feedback form, with a bounded feedback function $\alpha$ that is bounded in time and Lipschitz continuous in *x*. If $f_0$ and *g* are twice continuously differentiable, then

$$\begin{aligned} \textrm{PoI} \ge \frac{1}{4C} {\mathbb {E}}\int _0^T |Y_t|^2 dt, \end{aligned}$$

where *C* is a constant which depends only on the model coefficients and

36 $$\begin{aligned} Y_t&= {{\mathbb {E}}} \left\{ \widetilde{{\mathbb {E}}} \left[ \partial _\mu g \left( \widetilde{X}_T^\mathrm{{MFC}}, \mu _T^\mathrm{{MFC}} \right) \left( X_T^\mathrm{{MFC}}\right) \right. \right. \nonumber \\&\quad \left. \left. + \int _t^T \partial _\mu f_0 \left( \widetilde{X}_s^\mathrm{{MFC}}, \mu _s^\mathrm{{MFC}} \right) \left( X_s^\mathrm{{MFC}} \right) ds \right] \, \Big \vert \, {{\mathcal {F}}}_t \right\} . \end{aligned}$$

Intuitively, Proposition 6 gives a lower bound that can be calculated only by solving the MFC problem.

#### Proof of Proposition 6

We start by noticing that, for a new bounded control process $\boldsymbol{\beta }$ and for any $\varepsilon \in {\mathbb {R}}^{{d}},$

$$\begin{aligned} \begin{aligned}&\frac{d}{d\varepsilon } \Bigl [ J \bigl ( {\boldsymbol{\alpha }}^\mathrm{{MFC}} + \varepsilon {\boldsymbol{\beta }} ; \mu ^{ {\boldsymbol{\alpha }}^\mathrm{{MFC}} + \varepsilon \boldsymbol{\beta }} \bigr ) - J \bigl ( {\boldsymbol{\alpha }}^\mathrm{{MFC}} + \varepsilon {\boldsymbol{\beta }} ; \mu ^{\mathrm{{MFC}} }\bigr ) \Bigr ]_{\Big \vert \varepsilon =0} \\&\quad ={{\mathbb {E}}} \widetilde{{\mathbb {E}}} \biggl [ \int _0^T \partial _\mu f_0 \bigl ( X_t^\mathrm{{MFC}}, \mu _t^\mathrm{{MFC}} \bigr )\bigl ( {\widetilde{X}}_t^\mathrm{{MFC}}\bigr ) \cdot \partial {\widetilde{X}}_t^\mathrm{{MFC}} dt\\&\qquad + \partial _\mu g \bigl ( X_T^\mathrm{{MFC}}, \mu _T^\mathrm{{MFC}} \bigr ) \bigl ( {\widetilde{X}}_T^\mathrm{{MFC}}\bigr ) \cdot \partial {\widetilde{X}}_T^\mathrm{{MFC}} \biggr ], \end{aligned} \end{aligned}$$

where $\partial \tilde{X}_t^\mathrm{{MFC}} = \int _0^t \tilde{\beta }_s ds,$ $t\in [0,T],$ with the convention in Sect. 2.1, $\tilde{\boldsymbol{\beta }}$ is an independent copy of $\boldsymbol{\beta }$. We can compute the above derivative following the steps of the proof of the stochastic maximum principle (see e.g. [15, Chapters 3 & 6]). Since $\boldsymbol{\beta }$ is an arbitrary progressively measurable process, we can utilize Fubini’s theorem to rewrite the above identity as

37 $$\begin{aligned} \begin{aligned}&\frac{d}{d\varepsilon } \Bigl [ J \bigl ( {\boldsymbol{\alpha }}^\mathrm{{MFC}} + \varepsilon {\boldsymbol{\beta }} ; \mu ^{ {\boldsymbol{\alpha }}^\mathrm{{MFC}} + \varepsilon \boldsymbol{\beta }} \bigr ) - J \bigl ( {\boldsymbol{\alpha }}^\mathrm{{MFC}} + \varepsilon {\boldsymbol{\beta }} ; \mu ^{\mathrm{{MFC}} }\bigr ) \Bigr ]_{\Big \vert \varepsilon =0} \\&\quad ={{\mathbb {E}}} \widetilde{{\mathbb {E}}} \biggl [ \int _0^T \beta _t \biggl ( \partial _\mu g \bigl ( {\widetilde{X}}_T^\mathrm{{MFC}}, \mu _T^\mathrm{{MFC}} \bigr ) \bigl ( X_T^\mathrm{{MFC}}\bigr ) + \int _t^T \partial _\mu f_0 \bigl ( {\widetilde{X}}_s^\mathrm{{MFC}}, \mu _s^\mathrm{{MFC}} \bigr )\bigl ( X_s^\mathrm{{MFC}}\bigr ) ds \biggr ) dt \biggr ] \\&\quad = {\mathbb {E}}\int _0^T Y_s \cdot \beta _s ds, \end{aligned} \end{aligned}$$

with $(Y_t)_{t \in [0,T]}$ as in (36). Since ${\boldsymbol{\alpha }}^\mathrm{{MFC}}$ is an optimizer for the MFC problem, we have $\frac{d}{d\varepsilon } J \bigl ( {\boldsymbol{\alpha }}^\mathrm{{MFC}} + \varepsilon {\boldsymbol{\beta }}; \mu ^{{\boldsymbol{\alpha }}^\mathrm{{MFC}} + \varepsilon \boldsymbol{\beta }}\bigr ) = 0$. Going back to (37), we obtain:

$$\begin{aligned} \begin{aligned}&\frac{d}{d\varepsilon } \Bigl [ J \bigl ( {\boldsymbol{\alpha }}^\mathrm{{MFC}} + \varepsilon {\boldsymbol{\beta }} ; \mu ^{\mathrm{{MFC}} }\bigr ) \Bigr ]_{\Big \vert \varepsilon =0} = -{\mathbb {E}}\int _0^T Y_s \cdot \beta _s ds. \end{aligned} \end{aligned}$$

Moreover, returning to (37) and writing the analogue of the formula but at some $\varepsilon >0$, we can see by using the Lipschitz property of the derivatives of $f_0$ and *g* that, for $\varepsilon \in [0,1]$

$$\begin{aligned} \Bigl \vert \frac{d}{d \varepsilon } \Bigl [ J\bigl ({\boldsymbol{\alpha }}^\textrm{MFC} + \varepsilon {\boldsymbol{\beta }};\mu ^{\textrm{MFC}} \bigr )\Bigr ] - \frac{d}{d\varepsilon } \Bigl [ J \bigl ( {\boldsymbol{\alpha }}^\mathrm{{MFC}} + \varepsilon {\boldsymbol{\beta }} ; \mu ^{\mathrm{{MFC}} }\bigr ) \Bigr ]_{\Big \vert \varepsilon =0} \Bigr \vert \le C {{\mathbb {E}}} \int _0^T \vert \beta _t \vert ^2 dt. \end{aligned}$$

Thus,

$$\begin{aligned} \begin{aligned}&J\bigl ({\boldsymbol{\alpha }}^\textrm{MFC} + {\boldsymbol{\beta }};\mu ^{\textrm{MFC}} \bigr ) \le J\bigl ({\boldsymbol{\alpha }}^\textrm{MFC} ;\mu ^{\textrm{MFC}} \bigr ) - {{\mathbb {E}}} \int _0^T Y_t \cdot \beta _t dt + C {{\mathbb {E}}} \int _0^T \vert \beta _t \vert ^2 dt. \end{aligned} \end{aligned}$$

We then minimize over ${\boldsymbol{\beta }}$ and get, for $\beta _t=\tfrac{1}{2C} Y_t$ for all $t \in [0,T]$,

$$\begin{aligned} \begin{aligned}&J\bigl ({\boldsymbol{\alpha }}^\textrm{MFC} + {\boldsymbol{\beta }};\mu ^{\textrm{MFC}} \bigr ) \le J\bigl ({\boldsymbol{\alpha }}^\textrm{MFC} ;\mu ^{\textrm{MFC}} \bigr ) - \tfrac{1}{4C} {{\mathbb {E}}} \int _0^T \vert Y_t \vert ^2 dt, \end{aligned} \end{aligned}$$

which says that $\textrm{PoI} \ge \tfrac{1}{4C} {{\mathbb {E}}} \int _0^T \vert Y_t \vert ^2 dt.$

### Subpopulation Deviation: *p*-Partial MFGs

The idea is now to look at a mixed population where, instead of a single player deviating, a fixed proportion, say $p \in [0,1]$, of the population is allowed to deviate from the control prescribed by the social planner (i.e., MFC optimal control) In this new setting, we assume that a proportion $(1-p)$ of the population still follows the MFC optimal control when everyone is assumed to be cooperative. The remaining proportion *p* of players minimize their individual costs, and the population reaches an equilibrium, which we call a *p*-partial mean field equilibrium.

#### Definition and First Results

Throughout this subsection, we consider the same dynamics as in (2), with the same cost as in (3) and (4). We start with the following definition, which corresponds to a game with a population consisting of a proportion *p* of non-cooperative players and a proportion $(1-p)$ of players who continue to use the original MFC optimal control:

##### Definition 5

We call $(\hat{\boldsymbol{\alpha }}^p, p \hat{\boldsymbol{\mu }}^p +(1-p) {\boldsymbol{\mu }}^\mathrm{{MFC}})$ a *p*-partial mean field (MF) equilibrium if

(i) ${\boldsymbol{\mu }}^\mathrm{{MFC}}$ is the flow of distributions resulting from the (original) social planner’s optimization,
(ii) ${\hat{\boldsymbol{\alpha }^p}}$ is the minimizer of $J(\boldsymbol{\alpha }; p\hat{\boldsymbol{\mu }}^p+(1-p){\boldsymbol{\mu }}^\mathrm{{MFC}})$ given fixed population distribution flow $p\hat{\boldsymbol{\mu }}^p+(1-p){\boldsymbol{\mu }}^\mathrm{{MFC}}$,
(iii) ${\hat{\mu ^p_t}}={\mathcal {L}}(X^{{\hat{\boldsymbol{\alpha }^p}}}_t)$ for all $t\in [0,T]$.

In this model, given any fixed $p \in [0,1]$, given a *p*-partial MF equilibrium $({\hat{\boldsymbol{\alpha }^p}}; p{\boldsymbol{\mu }}^{{\hat{\boldsymbol{\alpha }^p}}}+(1-p){\boldsymbol{\mu }}^\mathrm{{MFC}}\big )$, we define the two costs, ${\hat{J}}_p$ and $J^*_p$, as follows:

38 $$\begin{aligned} \begin{aligned} {\hat{J}}_p&:= J\big ({\hat{\boldsymbol{\alpha }^p}}; p{\boldsymbol{\mu }}^{{\hat{\boldsymbol{\alpha }^p}}}+(1-p){\boldsymbol{\mu }}^\mathrm{{MFC}}\big )\\ J^*_p&:=J\big (\boldsymbol{\alpha }^\mathrm{{MFC}}; p{\boldsymbol{\mu }}^{{\hat{\boldsymbol{\alpha }^p}}}+(1-p){\boldsymbol{\mu }}^\mathrm{{MFC}}\big ). \end{aligned} \end{aligned}$$

The quantity $J^*_p$ is the cost to an individual player that still follows the social planner, while $\hat{J}_p$ is the cost to a non-cooperative player. Notice that this problem is different from the $\lambda$-interpolated MFG problem introduced in Sect. 3.2.2, where each player’s cost involves a mixture of individual and collective distributions. In a *p*-partial MF equilibrium, each player is either following the MFC optimal control or optimizing her individual cost. Furthermore, note that the $(1-p)$ proportion of players following the social planner’s recommendation use a control which is socially optimal only in the case where $p=0$. Otherwise, their control is sub-optimal. By definition, the cost of MFC, $J^*$, is equal to $J^*_0$ and the cost of the representative player in a regular MFG, ${\hat{J}}:=J^{\boldsymbol{\mu }^\mathrm{{MFG}}}(\boldsymbol{\alpha }^\mathrm{{MFG}})$, is equal to ${\hat{J}}_1$.

##### Remark 7

Notice that, $J^*_0 - {\hat{J}}_0$ is equal to the PoI since $J^*_0$ is the cost of MFC and ${\hat{J}}_0$ is the cost of a single player using their best response given that everyone else still follows the control prescribed by the social planner. Moreover,

$$\begin{aligned} \hat{J}_0 \le J^* = J^*_0 \le \hat{J}_1. \end{aligned}$$

We emphasize that a *p*-partial MF equilibrium is an equilibrium of a standard MFG whose coefficients depend on $\boldsymbol{\mu }^\mathrm{{MFC}}$. Indeed, one can consider the MFG corresponding to the running cost and the terminal cost (instead of $f_0$ and *g* respectively):

$$ (x,\mu ) \mapsto f_0^{p}( x, p \mu +(1-p) \mu ^\mathrm{{MFC}} ), \quad (x,\mu ) \mapsto g^{p} \bigl ( x, p \mu + (1-p) \mu ^\mathrm{{MFC}} ), $$

where $\mu ^\mathrm{{MFC}}$ is fixed and known from the players who are optimizing their individual cost. If we denote by $({\hat{\boldsymbol{\alpha }}},{\hat{\boldsymbol{\mu }}})$ the MFG equilibrium resulting from these costs functions, then the *p*-partial MF equilibrium as defined in Definition 5 is: $(\hat{\boldsymbol{\alpha }}^p, p \hat{\boldsymbol{\mu }}^p +(1-p) {\boldsymbol{\mu }}^\mathrm{{MFC}}) = ({\hat{\boldsymbol{\alpha }}}, p \hat{\boldsymbol{\mu }} +(1-p) {\boldsymbol{\mu }}^\mathrm{{MFC}})$.

The following result shows that the *p*-partial mean field model is well-posed.

##### Theorem 7

Suppose Assumptions 1 and 2 hold. Then, for any $p \in [0,1]$, there exists at least one *p*-partial MF equilibrium. Moreover, the *p*-partial mean field equilibrium is unique if *p* is small enough (i.e., $p<\varepsilon _0$ for some $\varepsilon _0>0$ independent of the choice of the initial condition of $\mu ^\mathrm{{MFC}}$).

##### Proof of Theorem 7

By realizing the *p*-partial MF equilibrium is an equilibrium of a standard MFG whose coefficients depend on $\boldsymbol{\mu }^\mathrm{{MFC}}$, the existence proof follows from existence results for MFGs such as under Assumption 2.(*i*) through [15, Theorem 4.32] and under Assumption 2.(*ii*) through [15, Theorem 4.53].

For a small *p*, we prove uniqueness by showing contraction. Assume that $\hat{\boldsymbol{\alpha }}^p$ and $\hat{\boldsymbol{\beta }}^p$ are two *p*-partial MF equilibria and denote by ${\boldsymbol{\mu }}^{{\hat{\boldsymbol{\alpha }}}^p}$ and ${\boldsymbol{\mu }}^{{\hat{\boldsymbol{\beta }}}^p}$ the corresponding mean fields. Then, using the convex of structure of the Hamiltonian, we obtain

$$\begin{aligned} \begin{aligned} \frac{1}{2} {{\mathbb {E}}} \int _0^T \vert \hat{\alpha }_t^{p} - \hat{\beta }_t^{p} \vert ^2 dt&\le J\left( \hat{\boldsymbol{\alpha }}^{p}; p {\boldsymbol{\mu }}^{\hat{\boldsymbol{\beta }}^p} + (1-p) {\boldsymbol{\mu }}^\mathrm{{MFC}} \right) \\&\quad - J\left( \hat{\boldsymbol{\beta }}^{p}; p{\boldsymbol{\mu }}^{\hat{\boldsymbol{\beta }}^p} + (1-p) {\boldsymbol{\mu }}^\mathrm{{MFC}} \right) \\ \frac{1}{2} {{\mathbb {E}}} \int _0^T \vert \hat{\alpha }_t^{p} - \hat{\beta }_t^{p} \vert ^2 dt&\le J\bigl ( \hat{{\boldsymbol{\beta }}^{p}}; p {\boldsymbol{\mu }}^{\hat{\boldsymbol{\alpha }}^p} + (1-p) {\boldsymbol{\mu }}^\mathrm{{MFC}} \bigr )\\&\quad - J\bigl ( \hat{{\boldsymbol{\alpha }}^{p}}; p {\boldsymbol{\mu }}^{\hat{\boldsymbol{\alpha }}^p} + (1-p) {\boldsymbol{\mu }}^\mathrm{{MFC}} \bigr ). \end{aligned} \end{aligned}$$

By adding the two lines above, we write:

39 $$\begin{aligned}&{{\mathbb {E}}} \int _0^T \vert \hat{\alpha }_t^{p} - \hat{\beta }_t^{p} \vert ^2 dt \nonumber \\&\quad \le p \int _{{{\mathbb {R}}}^d} \Bigl [ g \bigl ( x, p \mu _T^{\hat{\boldsymbol{\beta }}^p} + (1-p) \mu _T^\mathrm{{MFC}} \bigr ) - g\bigl ( x, p \mu _T^{\hat{\boldsymbol{\alpha }}^p} + (1-p) \mu _T^\mathrm{{MFC}} \bigr ) \Bigr ] d \bigl ( \mu _T^{\hat{\boldsymbol{\alpha }}^p} - \mu _T^{\hat{\boldsymbol{\beta }}^p} \bigr )(x) \nonumber \\&\qquad + p \int _{0}^T \int _{{{\mathbb {R}}}^d} \Bigl [ f_0\bigl ( x, p \mu _t^{\hat{\boldsymbol{\beta }}^p} + (1-p) \mu _t^\mathrm{{MFC}} \bigr ) - f_0\bigl ( x, p \mu _t^{\hat{\boldsymbol{\alpha }}^p} + (1-p) \mu _t^\mathrm{{MFC}} \bigr ) \Bigr ]\nonumber \\&\qquad d \bigl ( \mu _t^{\hat{\boldsymbol{\alpha }}^p} - \mu _t^{\hat{\boldsymbol{\beta }}^p} \bigr )(x) dt. \end{aligned}$$

Here, we call $\varepsilon$ a Bernoulli(*p*) random variable, that is independent of the $\sigma$-field ${{\mathcal {F}}}_T$ (extending the probability space, we can always find such a variable). Then, letting $M_\nu := \nu \varepsilon X_T^{\hat{\boldsymbol{\alpha }}^p}+(1- \nu ) \varepsilon X_T^{\hat{\boldsymbol{\beta }}^p} + (1-\varepsilon ) X_T^\textrm{MFC}$,

40 $$\begin{aligned}&\int _{{{\mathbb {R}}}^d} \Bigl [ g \bigl ( x, p \mu _T^{\hat{\boldsymbol{\beta }}^p} + (1-p) \mu _T^\mathrm{{MFC}} \bigr ) - g\bigl ( x, p \mu _T^{\hat{\boldsymbol{\alpha }}^p} + (1-p) \mu _T^\mathrm{{MFC}} \bigr ) \Bigr ] d \bigl ( \mu _T^{\hat{\boldsymbol{\alpha }}^p} - \mu _T^{\hat{\boldsymbol{\beta }}^p} \bigr )(x) \nonumber \\&\quad = \int _{{{\mathbb {R}}}^d} \Bigl [ g\Bigl (x , {{\mathcal {L}}} \bigl ( \varepsilon X_T^{\hat{\boldsymbol{\beta }}^p} + (1-\varepsilon ) X_T^\textrm{MFC} \bigr ) \Bigr ) - g\Bigl ( x , {{\mathcal {L}}} \Bigl ( \varepsilon X_T^{\hat{\boldsymbol{\alpha }}^p} + (1-\varepsilon ) X_T^\textrm{MFC} \Bigr )\Bigr ) \Bigr ]\nonumber \\&\qquad d \bigl ( \mu _T^{\hat{\boldsymbol{\alpha }}^p} - \mu _T^{\hat{\boldsymbol{\beta }}^p} \bigr )(x)\nonumber \\&\quad = {{\mathbb {E}}} \int _0^1 \biggr \{ \int _{{\mathbb {R}}^d} \Bigl [ \partial _\mu g\Bigl ( x , {{\mathcal {L}}} \bigl (M_\nu \bigl ),M_\nu \Bigr ) \cdot \Bigl ( \varepsilon X_T^{\hat{\boldsymbol{\alpha }}^p} - \varepsilon X_T^{\hat{\boldsymbol{\beta }}^p} \Bigr ) \Bigr ] d \bigl ( \mu _T^{\hat{\boldsymbol{\alpha }}^p} - \mu _T^{\hat{\boldsymbol{\beta }}^p} \bigr )(x)\biggr \} d \nu \nonumber \\&\quad = \int _0^1 {{\mathbb {E}}} \widetilde{{\mathbb {E}}} \Bigl [ \partial _\mu g\Bigl ( {\widetilde{X}}_T^{\hat{\boldsymbol{\alpha }}^p} , {{\mathcal {L}}} \bigl ( M_\nu \bigr ), M_\nu \Bigr ) \cdot \Bigl ( \varepsilon X_T^{\hat{\boldsymbol{\alpha }}^p} - \varepsilon X_T^{\hat{\boldsymbol{\beta }}^p} \Bigr ) \Bigr ] d\nu \nonumber \\&\qquad - \int _0^1 {{\mathbb {E}}} \widetilde{{\mathbb {E}}} \Bigl [ \partial _\mu g\Bigl ( {\widetilde{X}}_T^{\hat{\boldsymbol{\beta }}^p} , {{\mathcal {L}}} \bigl (M_\nu \bigr ), M_\nu \Bigr ) \cdot \Bigl ( \varepsilon X_T^{\hat{\boldsymbol{\alpha }}^p} - \varepsilon X_T^{\hat{\boldsymbol{\beta }}^p} \Bigr ) \Bigr ] d\nu . \end{aligned}$$

Proceeding similarly with $f_0$ and using Assumption 1.(*ii*), we obtain

$$\begin{aligned} \begin{aligned} {{\mathbb {E}}} \int _0^T \vert \hat{\alpha }_t^{p} - \hat{\beta }_t^{p} \vert ^2 dt&\le C_T p {{\mathbb {E}}} \bigl [ \sup _{0 \le t \le T} \vert X_t^{\hat{\boldsymbol{\alpha }}^p} - X_t^{{\hat{\boldsymbol{\beta }^p}}} \vert ^2\bigr ], \end{aligned} \end{aligned}$$

where the constant $C_T$ depends on *T* but is independent of *p*. We complete the proof, observing that:

$$\begin{aligned} {{\mathbb {E}}} \left[ \sup _{0 \le t \le T} \vert X_t^{{\hat{\boldsymbol{\alpha }^p}}} - X_t^{{\hat{\boldsymbol{\beta }^p}}} \vert ^2\right] \le C_T {{\mathbb {E}}} \int _0^T \vert \hat{\alpha }_t^{p} - \hat{\beta }_t^{p} \vert ^2 dt, \end{aligned}$$

$$\square $$

The following result says that the 0-partial mean field equilibrium provides the best (individual) cost (among all the *p*-partial mean field equilibria). We stress that this is the only result for which we use a concavity condition.

##### Proposition 8

Suppose Assumptions 1 and 2 hold. Assume that $f_0$ and *g* are displacement concave in the measure argument (see definition of displacement convexity in Assumption 3). Let $p \in [0,1]$ and let $(\hat{{\boldsymbol{\alpha }}}^{p},p\hat{\boldsymbol{\mu }}^{p}+(1-p)\boldsymbol{\mu }^\textrm{MFC})$ be a *p*-partial MF equilibrium. Denote $\hat{J}_p = J(\hat{{\boldsymbol{\alpha }}}^{p},p\hat{\boldsymbol{\mu }}^{p}+(1-p)\boldsymbol{\mu }^\textrm{MFC})$. Then:

$$\begin{aligned} \hat{J}_0 \le (1-p) \hat{J}_0 + p J^{*} \le \hat{J}_p \le J^{*}_p. \end{aligned}$$

##### Proof of Proposition 8

We write

$$\begin{aligned} \begin{aligned} \hat{J}_p&=J\bigl (\hat{\boldsymbol{\alpha }}^{p};p \hat{\boldsymbol{\mu }}^p + (1-p) {\boldsymbol{\mu }}^\mathrm{{MFC}} \bigr ) \\&= \frac{1}{2} {{\mathbb {E}}} \int _0^T \vert \hat{\alpha }_t^{p} \vert ^2 dt + {{\mathbb {E}}} \biggl [ \int _0^T f_0\bigl (X_t^{\hat{\boldsymbol{\alpha }}^p}, p \hat{\mu }^p_t + (1-p) \mu _t^\mathrm{{MFC}} \bigr ) dt\\&\quad + g \bigl (X_T^{\hat{\boldsymbol{\alpha }}^p}, p \hat{\mu }^p_T + (1-p) \mu _T^\mathrm{{MFC}} \bigr ) \biggr ] \\&\ge \frac{1}{2} {{\mathbb {E}}} \int _0^T \vert \hat{\alpha }_t^{p} \vert ^2 dt + p {{\mathbb {E}}} \biggl [ \int _0^T f_0\bigl (X_t^{\hat{\boldsymbol{\alpha }}^p}, \hat{\mu }^p_t \bigr ) dt + g\bigl (X_T^{\hat{\boldsymbol{\alpha }}^p},\hat{\mu }^p_T \bigr )\biggr ] \\&\hspace{15pt} + (1-p) {{\mathbb {E}}} \biggl [ \int _0^T f_0\bigl (X_t^{\hat{\boldsymbol{\alpha }}^p}, \mu _t^\mathrm{{MFC}} \bigr ) dt + g \bigl (X_T^{\hat{\boldsymbol{\alpha }}^p}, \mu _T^\mathrm{{MFC}} \bigr ) \biggr ] \\&\ge p J^* + (1-p) J\bigl (\hat{\boldsymbol{\alpha }}^{0};{\boldsymbol{\mu }}^\mathrm{{MFC}}\bigr )\\ \end{aligned} \end{aligned}$$

where the first inequality comes from the concavity of $f_0$ and *g* in the measure argument and the last inequality comes from the fact that $J^*$ is the MFC optimum and $\hat{\boldsymbol{\alpha }}^0$ is the minimizer of $J(\cdot ; \boldsymbol{\mu }^\mathrm{{MFC}})$. Since $J^* \ge \hat{J}_0 = J(\hat{\boldsymbol{\alpha }}^{0};{\boldsymbol{\mu }}^\mathrm{{MFC}})$ and $J^{*}_p \ge \hat{J}_p$, the proof of the inequality is completed.

Intuitively, the result in Proposition 8 states that when there are only a few of deviating (i.e., non-cooperative) players they will incur a lower cost. For example, if there is only one negligible deviating player, this player will have the lowest possible expected cost. This result also says that for any $p\in [0,1)$ expected cost of deviating players is lower than the cost of players that still follows the (original) MFC solution. In this way, this result gives a mathematical understanding of the free-rider phenomenon that explains that some individuals can benefit off of the collaborative efforts of others.

#### The Case of Monotone Interactions

In this subsection, we further assume in addition to the conclusion of Theorem 7 that the two running and terminal costs $f_0$ and *g* are monotone in the sense of Lasry and Lions monotonicity, see (30). Under these settings, we give further well-posedness results related to *p*-partial MFGs.

##### Theorem 9

Let ${\boldsymbol{\mu }}^\mathrm{{MFC}}$ be a flow of distributions resulting from the MFC problem. Then, for any $p \in [0,1]$, there exists a unique *p*-partial MF equilibrium.

Furthermore, the path

$$ [0,1] \ni p \mapsto \bigl (X_t^{\hat{\boldsymbol{\alpha }}^p} \bigr )_{0 \le t \le T} \in L^2\bigl ( \Omega ;{{\mathcal {C}}}([0,T];{{\mathbb {R}}}^d)\bigr ) $$

is Lipschitz continuous, where the space ${{\mathcal {C}}}([0,T];{{\mathbb {R}}}^d)$ on the right-hand side is equipped with the supremum norm. In particular, the two cost mappings

$$[0,1] \ni p \mapsto \hat{J}_p, \quad \text {and} \quad [0,1] \ni p \mapsto J_p^*$$

are also continuous.

##### Proof Idea for Theorem 9

Recalling that a *p*-partial mean field equilibrium is a solution to the MFG driven by the two functions $(x,m) \mapsto f_0( x, (1-p) \mu ^\mathrm{{MFC}} + p m)$ and $(x,m) \mapsto g \bigl ( x, (1-p) \mu ^\mathrm{{MFC}} + pm)$, which are monotone, we invoke Lasry–Lions uniqueness criterion to guarantee that the *p*-partial mean field equilibrium is unique [12] and [15, Sect. 3.4]. The continuity proof is given in Appendix 1.

We claim that stronger regularity properties like differentiability of the mapping $p \mapsto (X_t^{\hat{\boldsymbol{\alpha }}^p})_{0 \le t \le T}$, like differentiability when the functions $f_0$ and *g* are sufficiently smooth and satisfy the Lasry-Lions monotonicity condition. For its proof, linearization techniques similar to the ones used in [17, 62, 75] in the analysis of the master equation for monotone MFGs should be employed. For the sake of brevity of this paper, this technical results are omitted.

##### Corollary 10

There exists $p^* \in [0,1]$ such that $\hat{J}_{p^*} = J^*$.

The proof of Corollary 10 follows from the application of the intermediate value theorem and the inequality $\hat{J}_0 \le J^* \le \hat{J}_1$.

By combining Theorem 9 and Corollary 10, there is a $p^*$ such that $\hat{J}_{p^*} = J^*$ where for $p\in [0, p^*]$,the non-cooperative players have lower cost than the original MFC optimal cost. This intuitively explains why free-riding^1^ happens by showing that it is beneficial for the players not to collaborate and do the best for themselves when there are not too many of them, i.e., mathematically, when *p* is lower than $p^*$. This also shows that when there are many non-cooperative players they are worse off than the original MFC optimal cost which states that the populations with too many non-collaborative players are at risk of worse outcomes.

**Example. ** In order to illustrate the relationship between MFG equilibrium, MFC solution, and *p*-partial MF equilibrium, we focus on a toy model with one-dimensional state and control and visualize ${\hat{J}}_p$ and $J^*_p$ with respect to *p*. Our model is similar to the examples treated in [14, 15, p. 278] and [16, Chapter 6]. We consider the following dynamics

$$\begin{aligned} dX_{t} = \alpha _{t} dt + dB_{t}, \end{aligned}$$

and the following expected cost:

$$\begin{aligned} J^{\boldsymbol{\mu }}(\boldsymbol{\alpha }) = {{\mathbb {E}}} \biggl [ \int _{0}^T \frac{1}{2} ( X_{t}^2 + \alpha _{t}^2) dt + \frac{1}{2} (X_{T} - q \bar{\mu }_{T})^2 \biggr ], \end{aligned}$$

where we put a bar on top of a measure or a random variable to denote its mean. Throughout this example, $X_{0}$ is some fixed $x_{0} \in \mathbb {R}$ and *q* is a constant parameter. Equilibrium characterization with the differential equation system results are provided in the Appendix 1 for the sake of brevity of the paper.

**Fig. 1:**
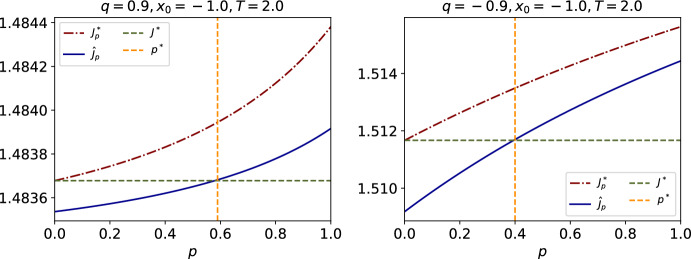
Costs of the non-cooperative players and the social planner followers are shown with (solid) blue and (dashed-dotted) red lines, respectively when $q=0.9$ (left) and $q=-0.9$ (right). The horizontal green line represents the cost of the MFC, i.e., social planner’s optimization. The vertical orange line represents the value of $p^*$ (Color figure online)

**Fig. 2:**
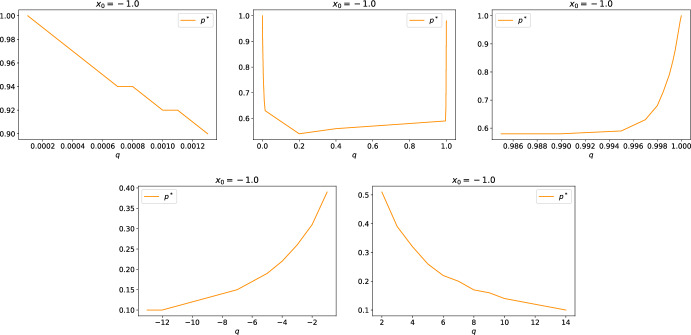
$p^*$ with respect to different *q* choices

In Fig. 1, under two different *q* choices ($q=0.9$ and $q=-0.9$), we see that $J_0^*$ corresponds to the MFC cost as expected, i.e., $J_0^*= J^*$. Furthermore, we see that ${\hat{J}}_0< J^{*}$, i.e., when a player deviates, their expected cost is lower. The PoI (see 4) can be seen as the difference between the green and blue curves at $p=0$. We also see that as *p* increases the cost for continuing to follow the original control prescribed by the social planner ($J^*_p$) increases. Moreover, we observe that the cost of non-cooperative players (${\hat{J}}_p$) is lower than the original social planner cost $J^*$ until $p^*$, which is the value of *p* where ${\hat{J}}_p = J^*$ (the value of *p* for which the blue and green lines cross, represented by a vertical dashed line). This is an instance of *free-ride* meaning that non-cooperative players can take advantage of cooperative players, but this advantage diminishes as the proportion of non-cooperative players increases. Figure 2 gives the variations of $p^*$ as a function of *q*.

### Repeated Deviations

We now consider the following repeated game: a large population starts behaving in a socially optimal way, but at each stage, some players decide to behave purely in their own interest, while others do not change their behavior. Players who are deviating from the social optimum can be allowed to repeatedly update their behavior (i.e., their control). We assume that the players are myopic in the sense that they do not know the proportion of *non-cooperative* players in the whole population, and they do not try to anticipate the population distribution in future iterations. They simply compute and implement a best response to the distribution they currently see, which is composed of non-cooperative and cooperative players. In this way, a sequence of distributions and a sequence of controls are generated.

Let us consider the dynamics and cost functions of Sect. 2. Recall we denote the mean field flow at the MFC optimum by $\mu ^\textrm{MFC} = (\mu ^\textrm{MFC}_t)_{t \in [0,T]}$. We define a sequence $(\mu ^n)_n$ of mean field flows by the following iterative procedure based on a sequence $Q_n \in [0,1]$, $n \ge 0$:

$$\begin{aligned} \mu ^{n+1}_t = Q_n \mu _t^\textrm{MFC} + (1-Q_n) \tilde{\mu }_t^{n+1}, \end{aligned}$$

where $(\tilde{\mu }^{n+1}_t)_{t\in [0,T]}$ is the mean field flow induced by the best response in environment $({\mu }^n_t)_{0 \le t \le T}$, namely $\tilde{\mu }^{n+1}_t$ is the law of $\tilde{X}_t^{n+1}$, where

41 $$\begin{aligned} \begin{aligned}&d \tilde{X}_t^{n+1} = - \tilde{Y}_t^{n+1} dt + \sigma dB_t, \\&d \tilde{Y}_t^{n+1} =- \partial _x f_0\left( \tilde{X}_t^{n+1},\mu _t^{n}\right) dt + Z_t^{n+1} dB_t, \\&{\tilde{Y}}_T^{n+1} = \partial _x g\left( \tilde{X}_T^{n+1},\mu _T^n\right) . \end{aligned} \end{aligned}$$

#### Theorem 11

Consider a sequence of proportions $p_i \in [0,1]$ for all $i\ge 0$. Let $p^* = 1-\prod _{i=0}^\infty (1-p_i)$. Let $Q_n = \prod _{i=0}^n(1-p_i)$, $n \ge 0$. Then,

(i) If $\mu ^{n}$ converges, its limit $\hat{\mu }^{p^*}$ is the *p*-partial mean field equilibrium with $p = p^*$, i.e., $\mu ^{p^*} = (1-p^*) \mu ^\mathrm{{MFC}} + p^* \mu ^{p^*\mathrm -MFG}$.
(ii) If *T* or the Lipschitz constants of $f_0$ and *g* are small enough, then $\mu ^{n}$ converges as $n\rightarrow \infty$.

#### Proof of Theorem 11

**Proof of** (*ii*) **(convergence): ** Before proving (*i*), we start with the proof of (*ii*) and split it into three steps.

**Step 1 (uniform bound). ** By item (*i*) in Assumption 2, FBSDE theory gives a uniform bound in $L^\infty$ for the processes $(\tilde{Y}_t^{n})_{t\in [0,T]}$. We then deduce that the functions $[0,T] \ni t \mapsto \mu _t^n$, $n \ge 0,$ which belong to ${{\mathcal {C}}}([0,T], {{\mathcal {P}}}_2({{\mathbb {R}}}^d)),$ form a relatively compact set.

**Step 2 (Cauchy property for** $(Q_k)_k$**). ** For later use, we note that since $Q_{k}$ converges towards $1-p^*$ as $k \rightarrow +\infty$, it is Cauchy. Hence: for any $\delta >0$, there exists $N_1(\delta )$ such that for all $k \ge N_1(\delta )$ and all $m \ge 0$, $|Q_{k+m} - Q_{k}| < \delta$.

**Step 3 (Cauchy property for** $\mu ^n$**). ** Recall that $W_1$ denotes the 1-Wasserstein distance. We write

$$\begin{aligned} \begin{aligned} W_1\bigl ( \mu _t^{n+m}, \mu _t^n \bigr )&\le C_1 \vert Q_{n-1} - Q_{n+m-1} \vert \\&\quad + \sup _{\Vert f \Vert _{1,\infty } \le 1} \int _{{{\mathbb {R}}}^d} f(x) d\Bigl ( (1-Q_{n-1}) \tilde{\mu }_t^{n} - (1-Q_{m+n-1}) \tilde{\mu }_t^{m+n} \Bigr )(x), \end{aligned} \end{aligned}$$

where $C_1 = \sup _{0 \le t \le T} \int _{{{\mathbb {R}}}^d} \vert x \vert d \mu _t^\textrm{MFC}(x)$. Then, denoting $C_2 = \sup _{n \ge 0} \sup _{0 \le t \le T} \int _{{{\mathbb {R}}}^d} \vert x \vert d \mu ^{n}_t(x)$, we obtain

$$\begin{aligned} W_1\bigl ( \mu _t^{n+m}, \mu _t^n \bigr )&\le (C_1 + C_2) \vert Q_{m+n-1} - Q_{n-1} \vert \\&\quad + (1-Q_{n-1}) \sup _{\Vert f \Vert _{1,\infty } \le 1} \int _{{{\mathbb {R}}}^d} f(x) d\Bigl ( \tilde{\mu }_t^{n} - \tilde{\mu }_t^{m+n} \Bigr )(x), \end{aligned}$$

and it follows,

42 $$\begin{aligned} \begin{aligned} W_1\left( \mu _t^{n+m}, \mu _t^n \right)&\le (C_1 + C_2) \vert Q_{m+n-1} - Q_{n-1} \vert + (1-Q_{n-1}) {{\mathbb {E}}} \left[ \vert \tilde{X}_t^{n+m} - \tilde{X}_t^{n} \vert \right] . \end{aligned} \end{aligned}$$

Next, we estimate ${{\mathbb {E}}} \bigl [ \vert \tilde{X}_t^{n+m} - \tilde{X}_t^{n} \vert \bigr ].$ We use the non-degeneracy of $\sigma$ and the fact that ${\tilde{Y}}_t^{n} = \theta _t^n({\tilde{X}}_t^n),$ where $\theta ^n$ is the solution of the PDE:

$$\begin{aligned} \partial _t \theta ^n_t(x) + \tfrac{1}{2} \textrm{Trace} \Bigl ( \sigma \sigma ^\top D^2_x \theta ^n_t(x) \Bigr ) - D_x \theta ^n_t(x) \theta ^n_t(x) + \partial _x f_0(x,\mu _t^{n-1}) = 0, \end{aligned}$$

with the boundary condition $\theta _T^n(x) = g(x,\mu _T^{n-1})$. By [64], we have a bound for $D_x \theta ^n$ that is independent of *n*. It is then possible to deduce that

$$\begin{aligned} \sup _{0 \le t \le T} {{\mathbb {E}}} \bigl [ \vert \tilde{X}_t^{n+m} - \tilde{X}_t^{n} \vert \bigr ] \le \Gamma \sup _{0 \le t \le T} {{\mathbb {E}}} \bigl [ \vert \theta ^{n} (t,\tilde{X}_t^{n+m}) - \tilde{Y}_t^{n} \vert \bigr ]. \end{aligned}$$

To control the right-hand side, we expand $d_t \theta ^{n}(t,\tilde{X}_t^{n+m})$ by means of Itô’s formula. This gives

$$\begin{aligned} \begin{aligned} d_t \left( \theta ^{n}(t,\tilde{X}_t^{n+m}) - \tilde{Y}_t^{n} \right)&= D_x\theta ^{n}(t,\tilde{X}_t^{n+m})\left( \theta ^{n}(t,\tilde{X}_t^{n+m}) - \tilde{Y}_t^{n} \right) dt \\&\qquad +\Bigl ( \partial _x f_0\bigl ( \tilde{X}_t^{n+m},\mu _t^{n+m-1}\bigr ) - \partial _x f_0\bigl ( \tilde{X}_t^{n+m},\mu _t^{n-1}\bigr ) \Bigr ) dt + dM_t, \end{aligned} \end{aligned}$$

where $(M_t)_{0 \le t \le T}$ is a martingale whose form does not matter for the estimate. At terminal time *T*, we have $\theta ^{n}(T,\tilde{X}_T^{n+m}) - \tilde{Y}_T^{n} = \partial _x g(\tilde{X}_T^{n+m},\mu _T^{n+m-1}) - \partial _x g(\tilde{X}_T^{n+m},\mu _T^{n-1})$. Fixing $t \in [0,T]$, integrating between *t* and *T* and taking conditional expectation given ${{\mathcal {F}}}_t$, we obtain

$$\begin{aligned} \begin{aligned} \bigl \vert \theta ^{n}(t,\tilde{X}_t^{n+m}) - \tilde{Y}_t^{n} \bigr \vert&\le c_0 W_1(\mu _T^{n-1},\mu _T^{n+m-1}) + c_0 \int _t^T W_1(\mu _s^{n-1},\mu _s^{n+m-1}) ds \\&\quad + \Gamma {{\mathbb {E}}} \biggl [ \int _t^T \bigl \vert \theta ^{n}(s,\tilde{X}_s^{n+m}) - \tilde{Y}_s^{n} \bigr \vert ds \, \vert \, {{\mathcal {F}}}_t \biggr ]. \end{aligned} \end{aligned}$$

Here, $c_0$ is the Lipschitz constant of the coefficients $f_0$ and *g* in the measure argument, while $\Gamma$ is the bound for the gradient of $\theta ^{n}$. Importantly, $\Gamma$ does not on depend on $c_0$. Taking expectation on both sides and applying Gronwall’s lemma, we obtain

$$\begin{aligned} \begin{aligned} {{\mathbb {E}}} \bigl \vert \theta ^{n}(t,\tilde{X}_t^{n+m}) - \tilde{Y}_t^{n} \bigr \vert \le c_0 (1+T) \exp (\Gamma T) \sup _{0 \le s \le T} W_1(\mu _s^{n-1},\mu _s^{n+m-1}). \end{aligned} \end{aligned}$$

Back to (42), we get

$$\begin{aligned} \begin{aligned} W_1\bigl ( \mu _t^{n+m}, \mu _t^n \bigr )&\le (C_1 + C_2) \vert Q_{m+n-1} - Q_{n-1} \vert \\&\quad + c_0 (1+T) \exp (\Gamma T) \sup _{0 \le s \le T} W_1(\mu _s^{n-1},\mu _s^{n+m-1}). \end{aligned} \end{aligned}$$

Denoting $C_3 = c_0 (1+T) \exp (\Gamma T)$, we note that if *T* is small enough or $c_0$ is small enough, then $C_3<1$. To conclude, we use induction. We have:

$$\begin{aligned} \begin{aligned} \sup _{0 \le s \le T} W_1\bigl ( \mu _s^{n+m}, \mu _s^n \bigr )&\le (C_1 + C_2) \vert Q_{m+n-1} - Q_{n-1} \vert + C_3 \sup _{0 \le s \le T} W_1(\mu _s^{n+m-1},\mu _s^{n-1}) \\&\le \underbrace{(C_1 + C_2) \sum _{i=1}^n C_3^{i-1} \vert Q_{m+n-i} - Q_{n-i} \vert }_{\boldsymbol{A}} + \underbrace{C_3^n \sup _{0 \le s \le T} W_1(\mu _s^{m},\mu _s^{0})}_{\boldsymbol{B}}. \end{aligned} \end{aligned}$$

For $\boldsymbol{B}$, note that it converges to 0 as *n* tends to infinity, thanks to the fact that $C_3<1$ and thanks to **Step 1** of this proof.

For $\boldsymbol{A}$, we can show that it converges to 0 as $n \rightarrow \infty$ as follows: we split it as

$$ \boldsymbol{A} = \underbrace{C_1+C_2 \sum _{i = 1}^{N_0} C_3^{i-1} |Q_{n+m-i} - Q_{n-i}|}_{\boldsymbol{A_1}} + \underbrace{C_1+C_2 \sum _{i=N_0+1}^n C_3 ^{i-1} |Q_{n+m-i} - Q_{n-i}|}_{\boldsymbol{A_2}}, $$

for any $N_0 \in \{1,\dots ,n\}$. One can choose $N_0$ large enough such that both $\boldsymbol{A_1}$ (using the fact that $C_3<1$) and $\boldsymbol{A_2}$ (using the fact that $|Q_{n+m-i} - Q_{n-i}| \le 1$ for $N_0$ large enough) are arbitrarily small.

We deduce that $(\mu ^n)_n$ form a Cauchy sequence.

**Proof of (***i***) (identification of the limit): ** Let $(\mu ^\infty _t)_{0 \le t \le T}$ be the limit. Following (41), we solve

$$\begin{aligned} \begin{aligned}&d \tilde{X}_t = - \tilde{Y}_t dt + \sigma dB_t, \\&d \tilde{Y}_t =- \partial _x f_0(\tilde{X}_t,\mu _t^{\infty }) dt + Z_t^{n+1} dB_t, \\&{\tilde{Y}}_T = \partial _x g(\tilde{X}_T\mu _T^\infty ). \end{aligned} \end{aligned}$$

We can prove, as above, that $\lim _{n \rightarrow \infty } {{\mathbb {E}}} \bigl [ \vert \tilde{X}_t^n - \tilde{X}_t \vert \bigr ]= 0.$ Recalling that

$$\begin{aligned} \mu ^{n+1}_t = Q_n \mu _t^\textrm{MFC} + (1-Q_n) \tilde{\mu }_t^{n+1} = Q_n \mu _t^\textrm{MFC} + (1-Q_n) {{\mathcal {L}}} \bigl ( \tilde{X}_t^{n+1} \bigr ), \end{aligned}$$

we deduce that $\mu _t^{\infty } = \bigl ( 1 - p^*\bigr ) \mu _t^\textrm{MFC} + p^* {{\mathcal {L}}} \bigl ( \tilde{X}_t \bigr ),$ i.e., we have found a $p^*$-mixed equilibrium.

We conclude our analysis of this iterative procedure with a special case of Theorem 11.

#### Corollary 12

Assume $p_i$ is constant w.r.t. *i*. If $\mu ^{n}$ converges, its limit is the MFG solution.

In this way, we conclude that even if players use myopic adjustments, they converge to the Nash equilibrium when they are *allowed* to deviate over iterations.

## Conclusion

In this paper, we analyze the bidirectional connections between MFGs and MFC. The first part of the paper discusses incentivization methods to coerce non-cooperative players into reaching a Nash equilibrium either having the same cost function or the same controls as in the (original) social optimum. In this part, we also introduce and theoretically analyze a new game, which we call $\lambda$-interpolated MFG, where each player’s objective is a mixture of individual and social costs. In the second part of the paper, we identify and quantify the instability of the MFC optimum (i.e., social optimum) with the notion of Price of Instability. We further introduce and theoretically analyze a new game called *p*-partial MFG, where a *p*-portion of the players are allowed to deviate from the social optimum. We conclude this part by analyzing a repeated game in which at each stage, the players have incomplete information about the game: they only see the whole distribution and they do not know the proportion *p* of deviating players. We further analyze the convergence of this iterative decision process.

## Appendix A: Additional Remarks on Sect. 3.1

### A.1. Remark on the Smoothness of *V*

We state typical assumptions under which the function *V* in (18) is known to be smooth. Typically, the Hamilton–Jacobi–Bellman equation associated with the MFC problem (17) has a classical solution *v* when the coefficients are smooth (w.r.t. the measure argument) and convex (also w.r.t. the measure argument). We refer for instance to the book [62]. In this latter work, convexity in the measure argument is understood in the space of measures. Another approach, considered for instance in the paper [75] (see also the more recent paper [76]) is to require the coefficients to be displacement convex, i.e., to ask their lifts on the space of probability measures to be convex, which is in line with Remark 1.

Once the value function *v* is known to be smooth, we can study the regularity of *V*. Below, we give an outline. The first observation is that, from (19),

$$\begin{aligned} \partial _\mu v(t,\mu )(x) = \partial _x V(t,x,\mu ) + \int _{{{\mathbb {R}}}^d} \partial _{\mu } V(t,x',\mu )(x) d\mu (x'), \qquad x \in {\mathbb {R}}^d. \end{aligned}$$

In turn, this says that the function $\bar{\alpha }$ in (22) is smooth. Then, we notice that (23) is in fact a linear PDE on the space of probability measures driven by the generator of the MKV SDE:

$$\begin{aligned} dX_t = \bar{\alpha }\bigl (t,X_t,{{\mathcal {L}}}(X_t) \bigr ) dt + dB_t, \quad t \in [0,T]. \end{aligned}$$

We then refer to [77] for a probabilistic approach to the regularity of the solution to the linear PDE associated with the above equation.

## Appendix B: Proofs of Sect. 4.2

### B.1. Continuity Proof of Theorem 9

#### Proof

We notice that (the complete version of) Theorem 7 holds true under the standing assumption. Therefore, we can duplicate the proof of (39). We get, for two different values $p,'p \in [0,1]$,

$$\begin{aligned} \begin{aligned}&{{\mathbb {E}}} \int _0^T \vert \hat{\alpha }_t^{p} - \hat{\alpha }_t^{p'} \vert ^2 dt \\&\quad \le \int _0^T \biggl [ \int _{{{\mathbb {R}}}^d} \bigl [ f_0\bigl ( x, (1-p') \mu _t^\mathrm{{MFC}} + p' \mu _t^{\hat{\boldsymbol{\alpha }}^{p'}} \bigr ) \\&\qquad - f_0\bigl ( x, (1-p) \mu _t^\mathrm{{MFC}} + p \mu _t^{\hat{\boldsymbol{\alpha }}^{p}} \bigr ) \bigr ] d \bigl ( \mu _t^{\hat{\boldsymbol{\alpha }}^{p}} - \mu _t^{\hat{\boldsymbol{\alpha }}^{p'}} \bigr ) (x) \biggr ] dt \\&\qquad + \int _{{{\mathbb {R}}}^d} \bigl [ g\bigl ( x, (1-p') \mu _T^\mathrm{{MFC}} + p' \mu _T^{\hat{\boldsymbol{\alpha }}^{p'}} \bigr )\\&\qquad - g\bigl ( x, (1-p) \mu _T^\mathrm{{MFC}} + p \mu _T^{\hat{\boldsymbol{\alpha }}^{p}} \bigr ) \bigr ] d \bigl ( \mu _T^{\hat{\boldsymbol{\alpha }}^{p}} - \mu _T^{\hat{\boldsymbol{\alpha }}^{p'}} \bigr ) (x) . \end{aligned} \end{aligned}$$

Writing $f_0( x, (1-p') \mu _t^\mathrm{{MFC}} + p' \mu _t^{\hat{\boldsymbol{\alpha }}^{p'}})$ in the form

$$\begin{aligned} \begin{aligned}&f_0 \bigl ( x, (1-p') \mu _t^\mathrm{{MFC}} + p' \mu _t^{\hat{\boldsymbol{\alpha }}^{p'}} \bigr ) \\&\quad = \Bigl [ f_0 \bigl ( x, (1-p') \mu _t^\mathrm{{MFC}} + p' \mu _t^{\hat{\boldsymbol{\alpha }}^{p'}} \bigr ) - f_0 \bigl ( x, (1-p) \mu _t^\mathrm{{MFC}} + p \mu _t^{\hat{\boldsymbol{\alpha }}^{p'}} \bigr ) \Bigr ] \\&\qquad + f_0 \bigl ( x, (1-p) \mu _t^\mathrm{{MFC}} + p \mu _t^{\hat{\boldsymbol{\alpha }}^{p'}} \bigr ), \end{aligned} \end{aligned}$$

and similarly for *g*, and then using the monotonocity property and following (40), we obtain

$$\begin{aligned} \begin{aligned}&{{\mathbb {E}}} \int _0^T \vert \hat{\alpha }_t^{p} - \hat{\alpha }_t^{p'} \vert ^2 dt \le C \vert p-p' \vert \sup _{0 \le t \le T} {{\mathbb {E}}} \left[ \vert X_t^{\hat{\boldsymbol{\alpha }}^p} - X_t^{\hat{\boldsymbol{\alpha }}^{p'}} \vert ^2\right] , \end{aligned} \end{aligned}$$

from which the Lipschitz property follows.

The second continuity result is a consequence of the latter computation, noticing that

$$\begin{aligned} \begin{aligned}&{{\mathbb {E}}} \int _0^T \vert \hat{\alpha }_t^{p} - \hat{\alpha }_t^{p'} \vert ^2 dt \le C \vert p-p' \vert ^2, \end{aligned} \end{aligned}$$

and using the fact that $f_0$ and *g* are locally Lipschitz as a consequence of Assumption 1.

### B.2. Equilibrium Characterizations for the Example Model in Sect. 4.2

Below we give the equilibrium characterization results for the MFG equilibrium, MFC solution, and *p*-partial MFGs. The proofs for MFG and MFC characterizations are similar to the proofs in [16] and are not presented for the sake of brevity. In order to be able to calculate the cost functionals directly, we will use the analytic approach that utilizes Hamilton–Jacobi–Bellman (HJB) and Kolmogorov-Fokker-Planck (KFP) equations.

#### MFG Equilibrium Characterization

In the MFG problem, the equilibrium control is given as $\alpha ^\mathrm{{MFG}}_t = -(X_t+r_t)$ and the cost is given as ${\hat{J}}_1 = \frac{1}{2}x_0^2 + r_0x_0+s_0$, where $(\boldsymbol{r},\boldsymbol{s}, \boldsymbol{{\bar{X}}})$ solve the following forward-backward ordinary differential equation (FBODE) system:

43 $$\begin{aligned} \left\{ \begin{aligned} \dot{r}_t&= r_t\quad r_T=-q{\bar{X}}_T\\ \dot{s}_t&= \dfrac{1}{2} (r_t^2-1)\quad s_T=\dfrac{1}{2}q^2{\bar{X}}_T^2\\ \dot{{\bar{X}}}_t&= -({\bar{X}}_t+r_t)\quad {\bar{X}}_0 = x_0. \end{aligned} \right. \end{aligned}$$

Notice that the solution satisfies, for all $t \in [0,T]$, $r_t = -q \bar{X}_T \exp (t-T)$, and $(1-qT)\bar{X}_T = e^{-T} \bar{X}_0$. This system has a unique solution if $qT \ne 1$.

The condition $qT \ne 1$ is satisfied in particular if $q<0$, which matches the requirements for the usual monotonicity condition.

#### MFC Solution Characterization

In the MFC problem, the optimal control is given as $\alpha ^\mathrm{{MFC}}_t = -(X_t+r_t)$ and the cost is given as $J^*_0 = \frac{1}{2}x_0^2 + r_0x_0+s_0+(1-q)q{\bar{X}}_T^2$, where $(\boldsymbol{r},\boldsymbol{s}, \boldsymbol{{\bar{X}}})$ solve the following FBODE system:

44 $$\begin{aligned} \left\{ \begin{aligned} \dot{r}_t&= r_t  &   r_T=-2q{\bar{X}}_T+q^2{\bar{X}}_T\\ \dot{s}_t&= \dfrac{1}{2} (r_t^2-1)  &   s_T=\dfrac{1}{2}q^2{\bar{X}}_T^2\\ \dot{{\bar{X}}}_t&= -({\bar{X}}_t+r_t) \qquad \qquad  &   {\bar{X}}_0 = x_0. \end{aligned} \right. \end{aligned}$$

Notice that the solution satisfies, for all $t \in [0,T]$, $r_t = (-2q+q^2) {\bar{X}}_T \exp (t-T)$, and $(1-(2q-q^2)T)\bar{X}_T = e^{-T} \bar{X}_0$. This system has a unique solution if $(2q-q^2)T \ne 1$.

Here again, the condition $(2q-q^2)T \ne 1$ is satisfied in particular if $q<0$, which matches the requirements for the usual monotonicity condition.

#### *p*-Partial MF Equilibrium Characterization

In the *p*-partial MFG, the equilibrium control is given as $\hat{\alpha }^{p}_t = -(X_t+r_t)$ and the cost is given as ${\hat{J}}_p = \frac{1}{2}x_0^2 + r_0x_0+s_0$, where $(\boldsymbol{r},\boldsymbol{s}, \boldsymbol{{\bar{X}}})$ implicitly depend on *p* and solve the FBODE system:

45 $$\begin{aligned} \left\{ \begin{aligned} \dot{r}_t&= r_t  &   r_T=-qp{\bar{X}}_T - q(1-p) {\bar{X}}_T^\mathrm{{MFC}}\\ \dot{s}_t&= \dfrac{1}{2} (r_t^2-1)  &   s_T=\dfrac{1}{2}\big (q p {\bar{X}}_T + q(1-p){\bar{X}}_T^\mathrm{{MFC}}\big )^2\\ \dot{{\bar{X}}}_t&= -({\bar{X}}_t+r_t) \qquad \qquad  &   {\bar{X}}_0 = x_0. \end{aligned} \right. \end{aligned}$$

Consistent with Remark 7, we notice that, when $p=1$, the *p*-partial mean field equilibrium characterized by (45) corresponds to the MFG equilibrium characterized by (43). Note that when $p=0$, the solution to (45) does not coincide with the solution to the MFC, (44). This is because, when $p=0$, (45) characterizes the optimal control for an infinitesimal non-cooperative player while the rest of the population is playing the MFC optimal control.

##### Proof of p-partial MF Equilibrium Characterization

Since the mean field interactions are only in the terminal cost, the solution approach to find the *p*-partial MFG is equivalent to finding the MFG equilibrium in a model where the terminal cost is given as $\frac{1}{2}\big (x - q(p{\bar{X}}_T + (1-p){\bar{X}}_T^\mathrm{{MFC}})\big )^2,$ where ${\bar{X}}_T^\mathrm{{MFC}}$ is the mean of state at time *T* in the MFC problem and it will be taken as exogeneously.

We start by recalling the Hamiltonian and its minimizer:

46 $$\begin{aligned} \begin{aligned} H(t, x, m , y, \alpha ) =&\alpha y+ \dfrac{1}{2}x^2 + \dfrac{1}{2}\alpha ^2\\ \alpha ^* :=&\mathop {\mathrm {arg\,min}}\limits _{\alpha } H(t, x, m, y, \alpha ) = -y \end{aligned} \end{aligned}$$

where we use *m* to denote the state distribution by following the convention in analytic approach. We denote with $H^*(t, x, m, y):= H(t, x, m, y, \alpha ^*) = \dfrac{1}{2} x^2 -\dfrac{1}{2}y^2$ the optimal Hamiltonian. Then the Hamilton–Jacobi–Bellman (HJB) equation becomes:

47 $$\begin{aligned} \begin{aligned} -\partial _t u(t,x)&= \dfrac{1}{2} \partial ^2_{xx} u(t,x) + H^*(t, x, m, \partial _x u(t,x)),\\ u(T,x)&= \dfrac{1}{2}\Big (x - q(p{\bar{X}}_T + (1-p){\bar{X}}_T^\mathrm{{MFC}})\Big )^2. \end{aligned} \end{aligned}$$

We introduce the following ansatz:

48 $$\begin{aligned} u(t,x) = \dfrac{1}{2} \eta _t x^2 + r_t x + s_t. \end{aligned}$$

Then, we have $\partial _x u(t, x) = \eta _t x + r_t$ and $\partial _{xx}^2 u(t,x) = r_t$. By plugging (48) into (47), we obtain the following differential equations:

49 $$\begin{aligned} \begin{array}{llll} \dot{\eta }_t & = \eta _t^2-1 & &  \eta _T=1\\ \dot{r}_t & = \eta _t r_t & &  r_T=-qp{\bar{X}}_T - q(1-p) {\bar{X}}_T^\mathrm{{MFC}}\\ \dot{s}_t & = \dfrac{1}{2} (r_t^2-\eta _t) & &  s_T=\dfrac{1}{2}\big (q p {\bar{X}}_T + q(1-p){\bar{X}}_T^\mathrm{{MFC}}\big )^2. \end{array} \end{aligned}$$

Our next step is to write the KFP equation

50 $$\begin{aligned} \partial _t m(t, x) - \dfrac{1}{2} \partial ^2_{xx}m(t, x) + \nabla _x \big (-m(t, x) \partial _x u(t,x) \big )=0 \end{aligned}$$

We conclude by finding the dynamics of ${\bar{X}}_t$ can be found by using (50) and (48) as follows:

51 $$\begin{aligned} \begin{aligned} \dfrac{d{\bar{X}}_t}{dt}&= \dfrac{d}{dt} \int _{{\mathbb {R}}} x m(t, x)dx =\int _{{\mathbb {R}}} x \dfrac{\partial m(t, x)}{\partial t}dx\\&=\int _{{\mathbb {R}}} x\Big (\dfrac{1}{2} \partial ^2_{xx}m(t, x) - \nabla _x \big (-m(t, x) (\eta _t x+r_t) \big )\Big )dx\\&= -(\eta _t {\bar{X}}_t+r_t) \end{aligned} \end{aligned}$$

with $${\bar{X}}_0 = x_0$$.

After realizing $\eta _t=1$ for all $t\in [0,1]$, we end up with forward backward ordinary differential equation system and the *p*-partial MFG cost can be directly computed using the value function:

52 $$\begin{aligned} \begin{aligned} {\hat{J}}_p = \mathbb {E}\Big [u(0,x_0)\Big ] = \dfrac{1}{2} x_0^2 +r_0 x_0 + s_0. \end{aligned} \end{aligned}$$

## Appendix C: Notations

The list of notations can be found in Table 1. In Sect. 4, when $\boldsymbol{\alpha }^\mathrm{{MFC}}$ and $\boldsymbol{\mu }^\mathrm{{MFC}}$ notations are used, they represent the original MFC optimum control and mean field controlled by the original MFC optimum control.

**Table 1:** Notation

$J^{\boldsymbol{\mu }}(\boldsymbol{\alpha })= J(\boldsymbol{\alpha };\boldsymbol{\mu })$	Expected cost of the player using control $\boldsymbol{\alpha }$ given population distribution $\boldsymbol{\mu }$
$\hat{\alpha }(t,x,\mu ,y)$	Minimizer of Hamiltonian $H(t, x, \mu , y, \alpha )$ as in (6)
$\boldsymbol{\alpha }^{\mathrm{{MFC}}}$	MFC optimal control
$\boldsymbol{\alpha }^\mathrm{{MFG}}$	MFG equilibrium control
$\boldsymbol{X}^{\boldsymbol{\alpha }}$	State trajectory controlled by $\boldsymbol{\alpha }$
$\boldsymbol{X}^{\mathrm{{MFC}}}$	State trajectory controlled by $\boldsymbol{\alpha }^{\mathrm{{MFC}}}$, i.e., $\boldsymbol{X}^{\boldsymbol{\alpha }^{\mathrm{{MFC}}}}$
$\boldsymbol{X}^\mathrm{{MFG}}$	State trajectory controlled by $\boldsymbol{\alpha }^\mathrm{{MFG}}$, i.e., $\boldsymbol{X}^{\boldsymbol{\alpha }^\mathrm{{MFG}}}$
$\boldsymbol{\mu }^{\boldsymbol{\alpha }}$	Law of state trajectory (i.e., mean field) controlled by $\boldsymbol{\alpha }$
$\boldsymbol{\mu }^{\mathrm{{MFC}}}$	Law of state trajectory (i.e., mean field) controlled by $\boldsymbol{\alpha }^{\mathrm{{MFC}}}$
$\boldsymbol{\mu }^\mathrm{{MFG}}$	Law of state trajectory (i.e., mean field) controlled by $\boldsymbol{\alpha }^\mathrm{{MFG}}$
$J^{\lambda ,\textrm{MF}}( \boldsymbol{\alpha };{\boldsymbol{\mu }} )$	Expected $\lambda$-interpolated mean field cost of the player using control $\boldsymbol{\alpha }$ given population distribution $\boldsymbol{\mu }$
$\boldsymbol{\alpha }^\lambda$	$\lambda$-interpolated mean field equilibrium control
$\boldsymbol{X}^{\lambda }$	State trajectory controlled by $\boldsymbol{\alpha }^{\lambda }$
$\boldsymbol{\mu }^{\lambda }$	Law of state trajectory (i.e., mean field) controlled by $\boldsymbol{\alpha }^{\lambda }$
$\hat{\boldsymbol{\alpha }}^p$	*p*-partial mean field equilibrium control
$\hat{J}_p$	Expected cost of the non-cooperative player at the equilibrium when a proportion *p* of the population is deviating
$$J^*_p $$	Expected cost of the player continuing to follow the social planner at the equilibrium when a proportion *p* of the population is deviating
$\hat{J}_1$	Expected cost of representative player in MFG equilibrium
$J^* = J^\mathrm{{MFC}}(\boldsymbol{\alpha }^\mathrm{{MFC}})=J^*_0$	Expected cost of a player under social planner’s regulation (MFC optimum cost)

## Footnotes

1 In social sciences, the free rider problem is defined as the market failure that occurs when those who benefit from resources underpay. In our setup, it refers to the fact that the cost of non-cooperative players being less than the cooperative setup.
